# Ion‐channel degeneracy: Multiple ion channels heterogeneously regulate intrinsic physiology of rat hippocampal granule cells

**DOI:** 10.14814/phy2.14963

**Published:** 2021-08-02

**Authors:** Poonam Mishra, Rishikesh Narayanan

**Affiliations:** ^1^ Cellular Neurophysiology Laboratory Molecular Biophysics Unit Indian Institute of Science Bangalore India

**Keywords:** degeneracy, HCN channel, hippocampus, intrinsic excitability, inward rectifier potassium channel, patch‐clamp electrophysiology, persistent sodium channel

## Abstract

Degeneracy, the ability of multiple structural components to elicit the same characteristic functional properties, constitutes an elegant mechanism for achieving biological robustness. In this study, we sought electrophysiological signatures for the expression of ion‐channel degeneracy in the emergence of intrinsic properties of rat hippocampal granule cells. We measured the impact of four different ion‐channel subtypes—hyperpolarization‐activated cyclic‐nucleotide‐gated (HCN), barium‐sensitive inward rectifier potassium (K_ir_), tertiapin‐Q‐sensitive inward rectifier potassium, and persistent sodium (NaP) channels—on 21 functional measurements employing pharmacological agents, and report electrophysiological data on two characteristic signatures for the expression of ion‐channel degeneracy in granule cells. First, the blockade of a specific ion‐channel subtype altered several, but not all, functional measurements. Furthermore, any given functional measurement was altered by the blockade of many, but not all, ion‐channel subtypes. Second, the impact of blocking each ion‐channel subtype manifested neuron‐to‐neuron variability in the quantum of changes in the electrophysiological measurements. Specifically, we found that blocking HCN or Ba‐sensitive K_ir_ channels enhanced action potential firing rate, but blockade of NaP channels reduced firing rate of granule cells. Subthreshold measures of granule cell intrinsic excitability (input resistance, temporal summation, and impedance amplitude) were enhanced by blockade of HCN or Ba‐sensitive K_ir_ channels, but were not significantly altered by NaP channel blockade. We confirmed that the HCN and Ba‐sensitive K_ir_ channels independently altered sub‐ and suprathreshold properties of granule cells through sequential application of pharmacological agents that blocked these channels. Finally, we found that none of the sub‐ or suprathreshold measurements of granule cells were significantly altered upon treatment with tertiapin‐Q. Together, the heterogeneous many‐to‐many mapping between ion channels and single‐neuron intrinsic properties emphasizes the need to account for ion‐channel degeneracy in cellular‐ and network‐scale physiology.

## Graphical abstract

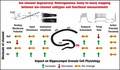

## 1 INTRODUCTION

Robust maintenance of neuronal intrinsic excitability and associated electrophysiological characteristics is critical to neuronal and network physiology, as perturbations to these properties result in pathological conditions (Beck & Yaari, 2008; Kullmann & Waxman, 2010; Marder & Goaillard, 2006; Nelson & Turrigiano, 2008; O'Leary, 2018; Poolos & Johnston, 2012; Rathour & Narayanan, 2019; Terzic & Perez‐Terzic, 2010; Turrigiano, 2011). A central question that spans neuronal subtypes is on how neurons achieve such robustness in maintaining their signature electrophysiological properties, despite the widespread expression of heterogeneities in biophysical properties. An elegant answer to this question arises from the degeneracy framework (Edelman & Gally, 2001), which postulates that disparate structural combinations could elicit similar function. With reference to the specific question on neuronal intrinsic characteristics, computational studies have pointed to the expression of degeneracy in terms of disparate ion channel combinations yielding characteristic cellular‐scale physiological signatures in different cell types, a phenomenon that has been referred to as ion‐channel degeneracy (Achard & De Schutter, 2006; Alonso & Marder, 2019; Das et al., 2017; Drion et al., 2015; Gjorgjieva et al., 2016; Goaillard & Marder, 2021; Gunay et al., 2008; Marder, 2011; Marder & Goaillard, 2006; Migliore et al., 2018; Mishra & Narayanan, 2019; Mittal & Narayanan, 2018; O'Leary, 2018; Onasch & Gjorgjieva, 2020; Rathour & Narayanan, 2012a, 2014, 2019; Taylor et al., 2009). In this study, employing dentate gyrus granule cells as the substrate, we electrophysiologically tested the expression of ion‐channel degeneracy in the emergence of cellular‐scale physiology. We employed a constellation of intrinsic electrophysiological measurements as the functional readouts and four distinct subthreshold activated ion channels as the structural elements and searched for signatures that point to the expression of ion‐channel degeneracy.

To sustain degeneracy, it is imperative that several structural components be capable of regulating any given function. Thus, in systems manifesting degeneracy, perturbation to several structural components should alter function, thereby precluding explicit one‐to‐one relationships between individual structural components and functional outcomes. Hence, a principal signature for the expression of degeneracy is the ability of multiple structural components to regulate a functional outcome. Another signature of the expression of degeneracy in systems showing same functional outcomes is the manifestation of parametric variability in the underlying structural components. Specifically, as different combinations of structural components can elicit the same function, the expression profiles of individual structural components would be variable. This implies that, in systems expressing degeneracy, perturbation of any single structural component would elicit variable impact on functional outcomes. Consistent with this general framework, prior computational studies, involving ion‐channel knockouts or perturbations, have revealed important testable predictions that point to the expression of ion‐channel degeneracy in the emergence of cellular‐scale function (Basak & Narayanan, 2018, 2020; Beining et al., 2017; Drion et al., 2015; Jain & Narayanan, 2020; Marder, 2011; Marder & Goaillard, 2006; Mishra & Narayanan, 2019, 2021; Mittal & Narayanan, 2018; O'Leary, 2018; Onasch & Gjorgjieva, 2020; Rathour & Narayanan, 2014):

1. Knocking out or perturbing any single ion channel altered multiple cellular‐scale physiological measurements, and any specific cellular‐scale physiological measurement was altered by knocking out or perturbing several ion channels; and
2. The impact of blocking individual ion channels on cellular‐scale measurements is variable. Specifically, the expression of ion‐channel degeneracy translates to differential expression of individual ion‐channels in different neurons of the same subtype.

In earlier studies (Mishra & Narayanan, 2019, 2021), we had employed computational models to hypothesize the expression of ion‐channel degeneracy in the emergence of cellular‐scale function in the granule cells of the dentate gyrus (DG), with additional support from other computational studies on these cell types (Beining et al., 2017). In this study, we aimed to experimentally test this hypothesis by recording several cellular‐scale physiological measurements from DG granule cells using whole‐cell patch‐clamp electrophysiology, before and after treatment with distinct pharmacological agents that block four different subtypes of non‐inactivating subthreshold‐activated ion channels. We chose this class of subthreshold‐activated non‐inactivating ion channels because of the impact of these ion channels (across cell types) on a wide array of physiological measurements: resting, subthreshold, and suprathreshold. In testing our hypothesis on ion‐channel degeneracy, we assessed the manifestation of the two functional signatures for the expression of ion‐channel degeneracy mentioned above. These analyses were performed on 21 different physiological measurements covering resting, subthreshold, and suprathreshold aspects of neural function.

We found that hyperpolarization‐activated cyclic nucleotide‐gated cation channel (HCN), Ba‐sensitive inward rectifier potassium and persistent sodium (NaP) channels variably regulated action potential firing rate, a suprathreshold property of DG granule cells. Specifically, whereas blockade of HCN or Ba‐sensitive inward rectifier potassium (K_ir_) channels enhanced action potential firing rate, blockade of NaP channels reduced action potential firing rate. On the other hand, subthreshold measures of intrinsic excitability such as input resistance, temporal summation of currents mimicking synaptic inputs, and impedance amplitude were variably enhanced by blockade of HCN or Ba‐sensitive K_ir_ channels, but were not significantly altered by NaP channel blockade. As HCN and Ba‐sensitive K_ir_ channels manifest hyperpolarization‐dependent activation profiles, we delineated their independent contributions to electrophysiological measurements. To do this, we sequentially applied blockers of these two channel subtypes on the same cells, and confirmed that the actions of HCN and Ba‐sensitive K_ir_ channels independently altered sub‐ and suprathreshold properties of DG granule cells. Finally, we assessed the impact of tertiapin‐Q‐sensitive inward rectifier potassium channels on granule cells physiology, and found that none of the sub‐ or suprathreshold measurements were significantly altered upon treatment with tertiapin‐Q. Together, our results provide experimental evidence for a heterogeneous many‐to‐many mapping between ion channels and single‐neuron intrinsic properties, thereby electrophysiologically testing the postulate on the expression of ion‐channel degeneracy in DG granule cells.

## 2 MATERIALS AND METHODS

### 2.1 Ethical approval

All experiments reported in this study were performed in strict adherence to the protocols approved by the Institute Animal Ethics Committee (IAEC) of the Indian Institute of Science, Bangalore. Animals were provided ad libitum food and water and were housed with an automated 12‐h light–12‐h dark cycle, with the facility temperature maintained at 21 ± 2°C. All animals were obtained from the in‐house breeding setup at the central animal facility of the Indian Institute of Science. Surgical and electrophysiological procedures were similar to previously established protocols (Ashhad & Narayanan, 2016; Mishra & Narayanan, 2020; Narayanan & Johnston, 2007, 2008; Rathour et al., 2016) and are detailed below.

### 2.2 Slice preparation for in‐vitro patch‐clamp recording

Male Sprague–Dawley rats of 6–8 weeks age were anesthetized by intraperitoneal injection of a ketamine–xylazine mixture. After onset of deep anesthesia, assessed by cessation of toe‐pinch reflex, transcardial perfusion of ice‐cold cutting solution was performed. The cutting solution contained 2.5 mM KCl, 1.25 mM NaH_2_PO_4_, 25 mM NaHCO_3_, 0.5 mM CaCl_2_, 7 mM MgCl_2_, 7 mM dextrose, 3 mM sodium pyruvate, and 200 mM sucrose (pH 7.3, ~300 mOsm) saturated with 95% O_2_ and 5% CO_2_. Thereafter, the brain was removed quickly and 350‐μm thick near horizontal slices were prepared from middle hippocampi (Bregma, –6.5 mm to –5.1 mm), using a vibrating blade microtome (Leica Vibratome), while submerged in ice‐cold cutting solution saturated with 95% O_2_ and 5% CO_2_. The slices were then incubated for 10–15 min at 34°C in a chamber containing the holding solution (pH 7.3, ~300 mOsm) with the composition of: 125 mM NaCl, 2.5 mM KCl, 1.25 mM NaH_2_PO_4_, 25 mM NaHCO_3_, 2 mM CaCl_2_, 2 mM MgCl_2_, 10 mM dextrose, and 3 mM sodium pyruvate saturated with 95% O_2_ and 5% CO_2_. The slices were kept in a holding chamber at room temperature for at least 45 min before the start of recordings.

### 2.3 Electrophysiology: Whole‐cell current‐clamp recording

For electrophysiological recordings, slices were transferred to the recording chamber and continuously perfused with carbogenated artificial cerebrospinal fluid (ACSF/extracellular recording solution) at a flow rate of 2–3 ml/min. All neuronal recordings were performed under current‐clamp configuration at physiological temperatures (32–35°C), achieved through an in‐line heater that was part of a closed‐loop temperature control system (Harvard Apparatus). The carbogenated ACSF contained 125 mM NaCl, 3 mM KCl, 1.25 mM NaH_2_PO_4_, 25 mM NaHCO_3_, 2 mM CaCl_2_, 1 mM MgCl_2_, and 10 mM dextrose (pH 7.3; ~300 mOsm). Slices were first visualized under a 10× objective lens to locate the granule cell layer in the crest sector of the dentate gyrus (Amaral et al., 2007; Mishra & Narayanan, 2020). Then, a 63× water immersion objective lens was employed to perform patch‐clamp recordings from DG granule cells in the crest sector, through a Dodt contrast microscope (Carl Zeiss Axioexaminer). Whole‐cell current‐clamp recordings were performed from visually identified dentate gyrus granule cell somata, using Dagan BVC‐700A amplifiers. Electrophysiological signals were low‐pass filtered at 5 kHz and sampled at 10–40 kHz. All data acquisition and analyses were performed using custom‐written software in Igor Pro (Wavemetrics).

Borosilicate glass electrodes with resistance between 2 and 6 MΩ (more often electrodes with ~4 MΩ electrode resistance were used) were pulled (P‐97 Flaming/Brown micropipette puller; Sutter) from thick glass capillaries (1.5 mm outer diameter and 0.86 mm inner diameter; Sutter) and used for patch‐clamp recordings. The pipette solution contained 120 mM K‐gluconate, 20 mM KCl, 10 mM HEPES, 4 mM NaCl, 4 mM Mg‐ATP, 0.3 mM Na‐GTP, and 7 mM K2‐phosphocreatine (pH 7.3 adjusted with KOH, osmolarity ~300 mOsm). Series resistance was monitored (once every 30 s) using a large hyperpolarizing pulse and compensated online using the bridge‐balance circuit of the amplifier. Experiments were excluded only if the initial resting membrane potential was more depolarized than –60 mV or if series resistance rose above 30 MΩ, or if there were fluctuations in temperature or ACSF flow rate during the course of the experiment. Unless otherwise stated, experiments were performed at the initial resting membrane potential (reported here as *V*_RMP_) of the cell. Voltages have not been corrected for the liquid junction potential, which was experimentally measured to be ~8 mV.

### 2.4 Pharmacological blockers

#### 2.4.1 Synaptic receptor blockers

Drugs and their concentrations used in the experiments were 10 µM 6‐cyano‐7‐nitroquinoxaline‐2,3‐dione (CNQX), an AMPA receptor blocker; and 10 µM (+) bicuculline and 10 µM picrotoxin, both GABA_A_ receptor blockers (all synaptic receptor blockers were purchased from Allied Scientific) in the bath solution. The experimenter was not blind to the specific pharmacological agent being employed.

#### 2.4.2 Voltage‐gated ion channel blockers

ZD7288 (20 µM; Allied Scientific) was employed to block HCN channels. BaCl_2_ (50 µM; Sigma Aldrich) or 0.3 µM tertiapin‐Q (Tocris Biosciences) was used to block specific subtypes of inward rectifier potassium channels. Riluzole (20 µM; Allied Scientific) was used to block persistent sodium channels.

**FIGURE 1:**
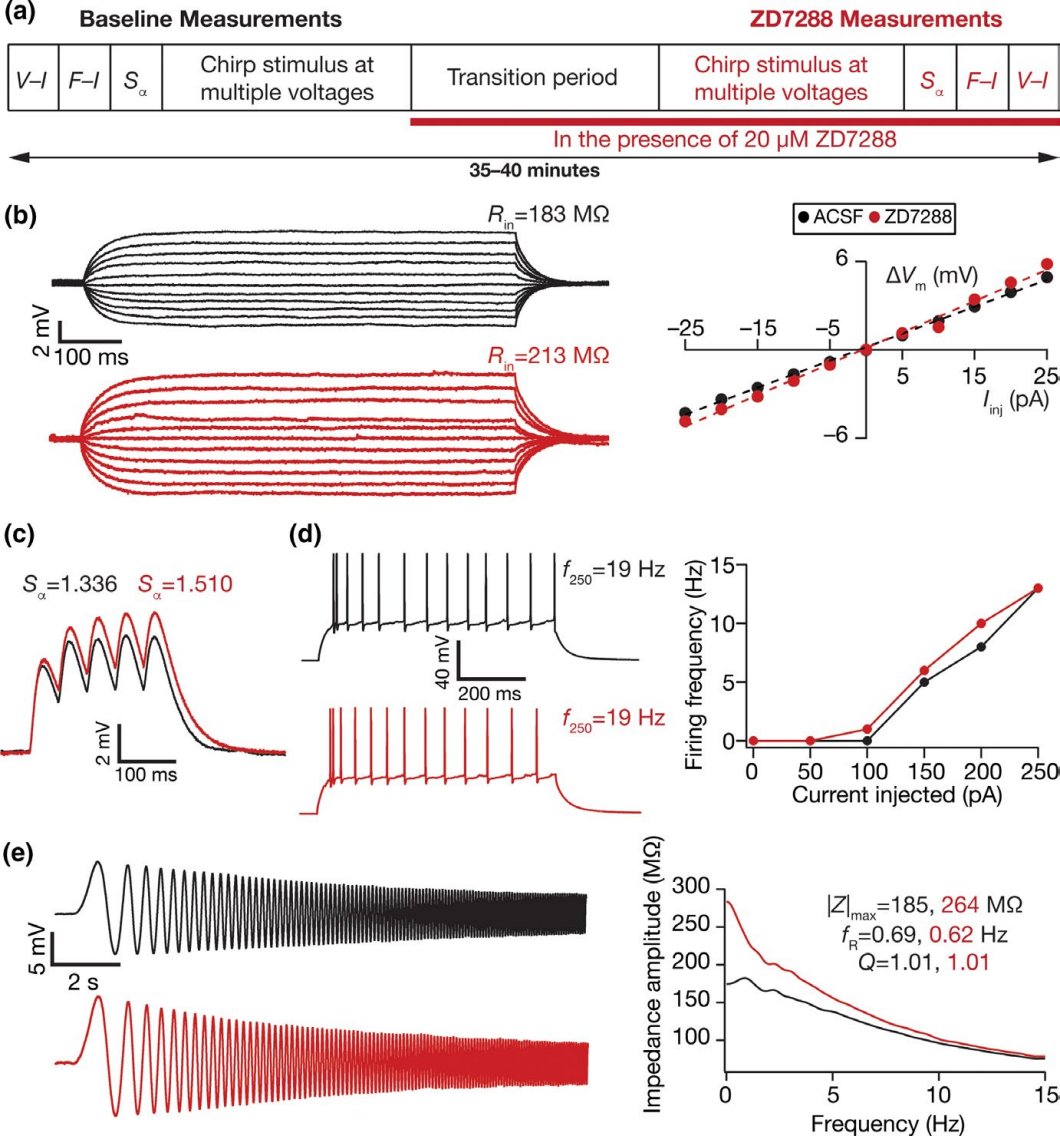
Illustration of the impact of acute treatment with ZD7288, a HCN channel blocker on the electrophysiological properties of DG granule cells. (a) Illustration of the protocol employed for assessing the impact of ZD7288 on granule cell physiology. (b) *Left*, Voltage responses of a DG granule cell to 700 ms current pulses of amplitude varying from −25 to +25 pA (in steps of 5 pA), with normal ACSF (*black*) and in the presence of 20 µM ZD7288 in the bath (*red*). *Right*, Input resistance (*R*_in_) was calculated as the slope of the *V–I* plot depicting steady‐state voltage response as a function of the injected current amplitude. (c) Voltage response of the example neuron to 5 alpha‐current injections arriving at 20 Hz, depicting temporal summation. Temporal summation ratio (*S*_α_) was computed as the ratio of the amplitude of the fifth response to that of the first. (d) *Left*, Voltage response of the example neuron to a 700‐ms current pulse of 250 pA in ACSF (*black*) and in the presence of ZD7288 (*red*). *Right*, Frequency of firing plotted as a function of injected current amplitude for the example cell. (e) *Left*, Voltage responses of the example neuron to the *Chirp15* current in ACSF (*black*) and in the presence of ZD7288 (*red*). *Right*, Impedance amplitude computed from the current stimulus shown in Figure 1b (*top*) and the voltage responses shown on the left. |*Z*|_max_ represents the maximum impedance amplitude, *Q* is resonance strength and resonance frequency is represented by *f*_R_. The experiment reported in this figure was performed in the presence of 10 µM CNQX, 10 µM (+) bicuculline and 10 µM picrotoxin. *V*_RMP_ = −79.8 mV for all traces and measurements depicted here

The protocols employed for measurements and the time course associated with the experiments are provided in appropriate figure panels associated with each (or each set of) pharmacological agents employed (e.g., Figure 1a for ZD7288 treatment). In performing time‐matched control experiments to evaluate potential drift in measurements, the protocol employed was similar to protocols employed with pharmacological agents, but without the application of any pharmacological agent. In these experiments, recordings were continued in the ACSF for both before and after measurements.

### 2.5 Quantification: Subthreshold measurements

We characterized subthreshold intrinsic properties of DG granule neurons using eight electrophysiological measurements obtained through several pulse current and frequency‐dependent current injections (Ashhad & Narayanan, 2016; Mishra & Narayanan, 2019, 2020; Narayanan & Johnston, 2007, 2008). The resting membrane potential (*V*_RMP_) was measured immediately after breaking the seal for whole‐cell recordings. Input resistance (*R*_in_) was measured as the slope of a linear fit to the steady‐state *V*–*I* plot obtained by injecting subthreshold current pulses of amplitudes spanning −25 to +25 pA, in steps of 5 pA (e.g., Figure 1b). Percentage sag was measured from the voltage response of the cell to a hyperpolarizing current pulse of 100 pA and was defined as 100 $(1‐V_{\text{ss}}/V_{\text{peak}}),$ where *V*_ss_ and *V*_peak_ depicted the steady‐state and peak voltage deflection from *V*_RMP_, respectively. To assess temporal summation, five α‐excitatory postsynaptic potentials (α–EPSPs) with 50 ms interval were evoked by current injections of the form $I_{\alpha }\left(t\right)=I_{\text{max}}t\exp\left(‐\alpha t\right)$, with α = 0.1 ms^−1^ (Figure 1c). Temporal summation ratio (*S*_α_) in this train of five EPSPs was computed as $E_{\text{last}}/E_{\text{first}}$, where *E*_last_ and *E*_first_ were the amplitudes of last and first EPSPs in the train, respectively.

The chirp stimulus used for characterizing the impedance amplitude (ZAP) profiles was a sinusoidal current of constant amplitude below firing threshold, with its frequency linearly spanning 0–15 Hz in 15 s (*Chirp15*). The magnitude of the ratio of the Fourier transform of the voltage response (e.g., Figure 1e) to the Fourier transform of the *Chirp15* stimulus formed the ZAP (e.g., Figure 1e):

$$\left|Z\left(f\right)\right|=\sqrt{{\left(\text{Re}\left(Z\left(f\right)\right)\right)}^{2}+{\left(\text{Im}\left(Z\left(f\right)\right)\right)}^{2}}$$

where $\text{Re}\left(Z\left(f\right)\right)$ and $\text{Im}\left(Z\left(f\right)\right)$ refer to the real and imaginary parts of the impedance *Z* as a function of frequency *f*. The maximum value of impedance across all frequencies was measured as the maximal impedance amplitude (|*Z*|_max_; Figure 1e). The frequency at which the impedance amplitude reached its maximum was the resonance frequency (*f*_R_). Resonance strength (*Q*) was measured as the ratio of the maximum impedance amplitude to the impedance amplitude at 0.5 Hz (Narayanan & Johnston, 2007). The impedance phase profile (ZPP) was computed as:

$$\phi =tan^{‐1}\frac{\text{Im}\left(Z\left(f\right)\right)}{\text{Re}\left(Z\left(f\right)\right)}$$

**FIGURE 2:**
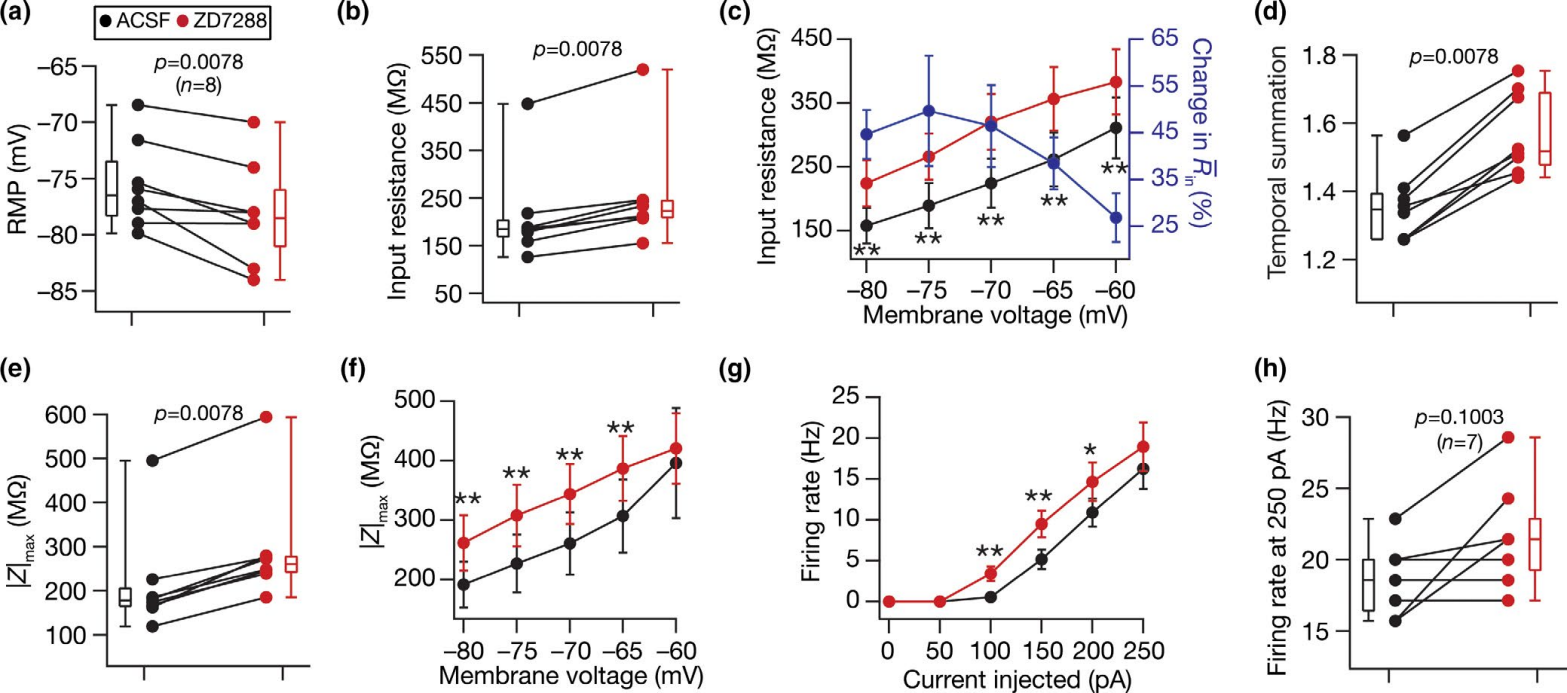
Acute treatment with ZD7288, a HCN channel blocker, hyperpolarized resting membrane potential and enhanced sub‐ and suprathreshold excitability of DG granule cells. Population data of measurements from DG granule cells recorded before and after adding ZD7288 to the bath: RMP (a); input resistance, *R*_in_ (b); membrane potential dependence of input resistance (c); temporal summation (d); impedance amplitude, |*Z*|_max_ (e) and its voltage dependence (f); firing rate at 0–250 pA current injection (g) and for 250 pA current injection (h). The Wilcoxon signed rank test was used for *p* value calculation in panels (a–b), (d–e) and (h), for comparing measurements from the same set of cells. For panel (c), (f) and (g), statistical comparisons were performed with paired Student's *t*‐test; *: *p* < 0.05, **: *p* < 0.005. All experiments reported in this figure were performed in the presence of 10 µM CNQX, 10 µM (+) bicuculline and 10 µM picrotoxin

Total inductive phase ($\Phi_{L}$) was defined as the area under the inductive part of the ZPP (Narayanan & Johnston, 2008). A crucial part of our experimental design is to obtain an input resistance estimate (computed using a 100‐pA hyperpolarizing current pulse) and impedance measurements at five different voltages (e.g., Figure 2c, Figure 2f). This was incorporated into our experimental design to assess if pharmacological treatment resulted in larger changes at hyperpolarized or depolarized voltages. These analyses were employed to gain insights about whether the action of the pharmacological agent is through channels that are activated by hyperpolarization or depolarization.

### 2.6 Quantification: Suprathreshold measurements

Suprathreshold physiological properties were computed using 13 different measurements. AP firing frequency was computed by extrapolating the number of spikes obtained during a 700 ms current injection to 1 s. Current amplitude of these pulse current injections was varied from 50 to 250 pA in steps of 50 pA, to construct the firing frequency versus injected current (*f–I*) plot (e.g., Figure 1d) at five different current injection values. Various AP related measurements (Mishra & Narayanan, 2019) were derived from the voltage response of the cell to a 250 pA pulse current injection. AP amplitude (*V*_AP_) was computed as the difference between the peak voltage of the spike ($V_{\text{AP}}^{\text{peak}}$) and *V*_RMP_. The temporal distance between the timing of the first spike and the time of current injection was defined as latency to first spike (*T*_1AP_). The duration between the first and the second spikes was defined as the first inter‐spike interval (*T*_1ISI_). AP half‐width (*T*_APHW_) was the temporal width measured at the half‐maximal points of the AP peak with reference to *V*_RMP_. The maximum ${\left.\frac{\mathrm{dV}}{\mathrm{dt}}\right|}_{\text{AP}}^{\text{max}}$, and minimum ${\left.\frac{\mathrm{dV}}{\mathrm{dt}}\right|}_{\text{AP}}^{\text{min}}$ values of the AP temporal derivative were calculated from the temporal derivative of the AP trace. The voltage in the AP trace corresponding to the time point at which the $\frac{\mathrm{dV}}{\mathrm{dt}}$ crossed 20 V/s defined AP threshold (*V*_th_). All suprathreshold measurements were obtained through current injections into the cell resting at *V*_RMP_.

### 2.7 Statistics

All statistical analyses were performed using the R computing package (http://www.r‐project.org/). In order to avoid false interpretations and to emphasize the heterogeneities, the entire range of measurements are reported in figures rather than providing only the summary statistics (Marder & Taylor, 2011; Rathour & Narayanan, 2019). Heterogeneities in the impact of individual pharmacological agents on electrophysiological measurements were quantified using three metrics of degree of variability (Tables 1, 2): standard deviation, interquartile range, and coefficient of variation. Across figures, the statistics employed for data presentation were consistent with the statistical test used to compare two populations of data. Specifically, when data are reported as mean ± SEM, parametric tests (paired or unpaired Student's *t*‐test) were employed, and when data are reported as median (along with the entire distribution of the data or the quartiles), we employed nonparametric tests (Wilcoxon ranked sum or signed rank tests). Results of statistical tests, with exact *p* values and the name of the statistical test employed, are provided in the figure panels or in the respective figure legends.

**TABLE 1:** Subthreshold measurements before and after drug (Control, ZD7288, BaCl_2_, Tertiapin Q, or Riluzole) treatment. Measurements are reported as before mean ± SEM and after mean ± SEM. Drug‐induced percentage changes in individual measurements are represented as mean ± SEM. Degree of variability in the percentage changes induced by each drug for each measurement are reported as standard deviation, interquartile range, and coefficient of variation. Resonance frequency ($f_{R}$) and resonance strength (*Q*) were around unity before or after treatment with any of the four pharmacological agents. Total inductive area ($\Phi_{L}$) was around zero across all conditions. $f_{R}$, *Q*, and $\Phi_{L}$ did not change significantly with any of the pharmacological agents. *n* = 10 for the Control group

Measurement	Symbol	Control	ZD7288	BaCl_2_		Tertiapin Q	Riluzole	
Subthreshold measurements (mean ± SEM before treatment; mean ±SEM after treatment; Percentage changes: mean ± SEM; standard deviation; interquartile range; coefficient of variation) *p* value from Wilcox rank sum test for comparison of values from ZD7288, BaCl_2_, tertiapin Q, and riluzole groups with respect to Control values.								
Resting membrane potential (mV) Kruskal–Wallis test across all groups: *p* = 6.9 × 10^−5^	*V*_RMP_	−75.71 ± 1.27; −75.33 ± 1.11; −0.48 ± 0.41; 1.10; 1.62; −2.31	−75.60 ± 1.36; −78.13 ± 1.60; 3.34 ± 0.90; 2.56; 3.12; 0.77 2.5 × 10^−3^	−77.18 ± 1.43; −73.71 ± 1.85; −4.56 ± 0.78; 2.07; 2.20; −0.45 1.7 × 10^−4^	−74.83 ± 1.14 −74.43 ± 1.41 −0.57 ± 0.48 1.27; 1.59; −2.22 0.75			−75.71 ± 1.27 −75.33 ± 1.11 −0.48 ± 0.41 1.10; 1.62; −2.31 0.83
Input resistance (MΩ) Kruskal–Wallis test across all groups: *p* = 1.7 × 10^−5^	*R*_in_	155.27 ± 4.34; 148.10 ± 10.17; −5.18 ± 4.24; 11.22; 10.85; −2.17	211.28 ± 35.05; 253.60 ± 39.33; 21.22 ± 2.77; 7.83; 14.10; 0.37 8.7 × 10^−4^	185.58 ± 14.94; 286.12 ± 23.10; 54.93 ± 5.10; 13.49; 6.84; 0.25 1.0 × 10^−4^	197.55 ± 27.85 187.71 ± 22.61 −3.52 ± 2.39 6.33; 7.14; −1.80 0.74			155.27 ± 4.34 148.10 ± 10.17 −5.18 ± 4.24 11.22; 10.85; −2.17 0.54
Temporal summation ratio Kruskal–Wallis test across all groups: *p* = 1.6 × 10^−5^	*S*_α_	1.24 ± 0.03; 1.24 ± 0.04; −0.40 ± 1.13; 2.77; 2.04; −6.87	1.35 ± 0.04; 1.57 ± 0.04; 16.21 ± 1.86; 5.25; 8.00; 0.32 3.1 × 10^−4^	1.32 ± 0.03; 1.71 ± 0.05; 29.91 ± 2.55; 6.75; 7.96; 0.23 5.8 × 10^−4^	1.34 ± 0.05 1.29 ± 0.03 −3.57 ± 1.97 5.22; 4.78; −1.46 1.0			1.23 ± 0.03 1.22 ± 0.04 −0.40 ± 1.13 2.77; 2.04; −6.87 0.29
Percentage sag Kruskal–Wallis test across all groups: *p* = 0.57	Sag	2.08 ± 0.44; 2.08 ± 0.32; 22.60 ± 26.31; 69.61; 96.05; 3.08	5.95 ± 3.00; 2.54 ± 0.54; −4.82 ± 34.61; 97.88; 84.51; −20.30 0.41	4.87 ± 1.40; 3.21 ± 0.85; −0.85 ± 51.28; 135.68; 67.98; −159.17 0.31	0.77 ± 0.06 0.67 ± 0.05 −7.18 ± 15.04 39.80; 19.63; −5.54 0.42			2.08 ± 0.44 2.08 ± 0.32 22.60 ± 26.31 69.61; 96.05; 3.08 0.81
Maximum impedance amplitude (MΩ) Kruskal–Wallis test across all groups: *p* = 3.9 × 10^−5^	|*Z*|_max_	158.67 ± 6.05; 168.75 ± 16.73; 6.07 ± 9.54; 25.23; 11.75; 4.16	213.77 ± 41.52; 292.49 ± 44.39; 42.27 ± 6.19; 17.50; 20.07; 0.41 4.6 × 10^−5^	182.33 ± 15.73; 311.87 ± 19.75; 73.09 ± 4.80; 12.70; 16.88; 0.17 1.0 × 10^−4^	187.15 ± 26.67 179.75 ± 19.47 −0.86 ± 4.76 12.60; 9.68; −14.65 0.54			158.67 ± 6.05 168.75 ± 16.73 6.07 ± 9.54 25.23; 11.75; 4.16 0.89

**TABLE 2:** Action potential measurements before and after drug (Control, ZD7288, BaCl_2_, Tertiapin Q or Riluzole) treatment. Measurements are reported as before mean ± SEM and after mean ± SEM. Drug‐induced percentage changes in individual measurements are represented as mean ± SEM. Degree of variability in the percentage changes induced by each drug for each measurement are reported as standard deviation, interquartile range, and coefficient of variation. *n* = 8 for the Control group

Measurement	Symbol	Control	ZD7288	BaCl_2_		Tertiapin Q	Riluzole	
Action potential measurements (mean ± SEM before treatment; mean ±SEM after treatment; Percentage changes: mean ± SEM; standard deviation; interquartile range; coefficient of variation) *p* value from Wilcox rank sum test for comparison of values from ZD7288, BaCl_2_, tertiapin Q, and riluzole groups with respect to Control values.								
Action potential threshold (mV) Kruskal–Wallis test across all groups: *p* = 0.48	*V*_th_	−38.49 ± 1.48; −40.81 ± 1.39; 6.30 ± 1.82; 5.16; 9.83; 0.82	−39.43 ± 1.02; −42.16 ± 0.86; 7.28 ± 2.80; 7.91; 8.71; 1.09 0.44	−40.08 ± 1.91; −41.86 ± 2.54; 4.26 ± 2.37; 6.28; 7.49; 1.47 0.69	−37.89 ± 1.35; −39.17 ± 1.60; 3.33 ± 1.66; 4.40; 3.29; 1.32 0.19			−38.49 ± 0.61; −37.87 ± 2.20; −1.68 ± 5.15; 11.53; 12.10; −6.84 0.1274
Peak action potential voltage (mV) Kruskal–Wallis test across all groups: *p* = 0.96	$V_{\text{AP}}^{\text{peak}}$	46.61 ± 1.84; 41.06 ± 3.66; −11.53 ± 8.28; 23.42; 13.09; −2.03	41.80 ± 3.26; 33.40 ± 4.01; −20.98 ± 4.67; 13.21; 12.16; −0.63 0.51	45.86 ± 2.03; 40.96 ± 2.70; −10.68 ± 4.12; 10.91; 14.21; −1.02 0.78	40.69 ± 2.13; 36.01 ± 1.82; −11.18 ± 3.06; 8.10; 15.05; −0.72 1.0			48.71 ± 1.37; 42.63 ± 3.43; −12.95 ± 4.65; 10.39; 13.37; −0.80 0.94
Action potential amplitude (mV) Kruskal–Wallis test across all groups: *p* = 0.97	*V*_AP_	127.73 ± 1.99; 122.31 ± 4.39; −4.26 ± 3.06; 8.64; 4.88; −2.03	117.81 ± 2.14; 109.69 ± 2.85; −6.91 ± 1.54; 4.35; 4.64; −0.63 0.96	123.71 ± 2.21; 118.42 ± 2.50; −4.24 ± 1.43; 3.79; 4.69; −0.89 0.96	115.33 ± 1.67; 110.98 ± 1.23; −3.70 ± 1.26; 3.34; 6.03; −0.90 1.0			125.12 ± 1.19; 118.70 ± 3.06; −5.18 ± 1.75; 3.92; 5.61; −0.76 0.72
Action potential halfwidth (ms) Kruskal–Wallis test across all groups: *p* = 0.13	*T*_APHW_	0.86 ± 0.03; 0.87 ± 0.03; 2.40 ± 3.58; 10.14; 9.77; 4.23	0.80 ± 0.03; 0.92 ± 0.09; 14.35 ± 7.70; 21.79; 26.52; 1.52 0.13	0.77 ± 0.03; 0.87 ± 0.03; 13.10 ± 4.21; 11.15; 11.74; 0.85 0.12	0.86 ± 0.04; 0.91 ± 0.04; 5.55 ± 1.16; 3.08; 3.09; 0.55 0.46			0.76 ± 0.02; 0.78 ± 0.06; 2.62 ± 6.12; 13.68; 23.81; 5.22 0.94
Peak dV/dt (V/s) Kruskal–Wallis test across all groups: *p* = 0.85	${\left.\frac{\mathrm{dV}}{\mathrm{dt}}\right|}_{\text{AP}}^{\text{max}}$	467.59 ± 25.10; 414.27 ± 35.59; −9.98 ± 9.65; 27.30; 20.45; −2.74	470.34 ± 44.87; 391.45 ± 48.91; −17.00 ± 5.29; 14.97; 10.12; −0.88 0.38	528.03 ± 24.98; 465.69 ± 40.89; −11.90 ± 5.87; 15.54; 15.55; −1.31 0.78	387.04 ± 20.69; 344.06 ± 13.54; −10.46 ± 3.13; 8.29; 12.12; −0.79 0.61			529.53 ± 29.00; 463.12 ± 73.12; −14.31 ± 8.49; 18.99; 26.58; −1.33 0.72
Minimum dV/dt (V/s) Kruskal–Wallis test across all groups: *p* = 0.18	${\left.\frac{\mathrm{dV}}{\mathrm{dt}}\right|}_{\text{AP}}^{\text{min}}$	−128.17 ± 3.91; −124.66 ± 4.86; −2.51 ± 3.48; 9.85; 12.27; −3.93	−133.05 ± 4.84; −111.92 ± 9.90; −16.26 ± 6.59; 18.63; 20.99; −1.15 0.33	−138.20 ± 2.58; −116.40 ± 4.85; −15.55 ± 4.05; 10.71; 10.59; −0.69 0.04	−120.32 ± 6.34; −114.13 ± 5.40; −4.90 ± 1.80; 4.76; 4.41; −0.97 0.54			−141.11 ± 5.69; −137.81 ± 11.08; −2.61 ± 5.32; 11.89; 15.68; −4.56 0.83
Latency to first spike (ms) Kruskal–Wallis test across all groups: *p* = 0.15	*T*_1AP_	72.93 ± 6.73; 56.16 ± 4.39; −20.94 ± 5.69; 16.09; 23.35; −0.77	36.46 ± 5.64; 24.18 ± 4.33; −29.39 ± 9.50; 26.87; 41.14; −0.91 0.72	35.33 ± 4.84; 25.43 ± 3.88; −30.31 ± 5.65; 14.96; 11.85; −0.49 0.54	38.65 ± 6.33; 37.99 ± 7.11; −4.30 ± 7.44; 19.68; 11.34; −4.57 0.09			45.37 ± 4.83; 49.38 ± 12.30; 5.33 ± 17.30; 38.68; 24.84; 7.26 0.13
First interspike interval (ms) Kruskal–Wallis test across all groups: *p* = 0.03	*T*_1ISI_	73.07 ± 24.66; 64.68 ± 22.18; −13.35 ± 14.87; 42.05; 41.31; −3.15	15.41 ± 4.86; 13.37 ± 5.59; 27.75 ± 50.55; 142.97; 60.07; 5.15 0.96	12.18 ± 3.00; 5.52 ± 0.47; −44.69 ± 7.66; 20.27; 24.51; −0.45 0.12	19.35 ± 6.40; 21.86 ± 7.87; 6.55 ± 11.36; 30.04; 28.37; 4.59 0.19			50.09 ± 17.38; 101.67 ± undef^a^; 86.94 ± undef^a^; undef^a^; undef^a^; undef^a^ undef^a^

^a^ undef: undefined. With Riluzole treatment, the number of action potentials elicited was low. All but one cell elicited a single action potential, making the computation of *T*_1ISI_ impossible (*T*_1ISI_ requires 2 action potentials). As there was only one *T*_1ISI_ measurement, SEM was undefined.

## 3 RESULTS

### 3.1 Blocking hyperpolarization‐activated cyclic‐nucleotide‐gated (HCN) nonspecific cation channels enhanced intrinsic excitability of DG granule cells

HCN channels contribute to prominent resting conductance in several neuronal subtypes and regulate intrinsic excitability, frequency‐dependent response properties, and temporal summation (Accili et al., 2002; Biel et al., 2009; Das et al., 2017; He et al., 2014; Magee, 2000; Pape, 1996; Robinson & Siegelbaum, 2003). We quantified the acute effect of ZD7288, a HCN channel blocker (BoSmith et al., 1993), on various physiological measurements of DG granule cells to quantitatively assess the impact of HCN channels on neuronal excitability and temporal summation. We performed whole‐cell current‐clamp recordings in ACSF and obtained baseline measurements along with characterizing membrane potential dependence profile. We then shifted the solution to 20 µM ZD7288 and after 10 min obtained the same set of measurements for comparing it with the measurements obtained in ACSF solution (Figure 1a). We observed enhancement in intrinsic excitability, reflected as an increase in input resistance (Figure 1b), temporal summation (Figure 1c), action potential firing rate in response to pulse current injections (Figure 1d) and an increase in impedance amplitude predominantly in the lower frequencies (Figure 1e).

When we repeated this protocol over a population of DG granule cells, we observed a significant hyperpolarization in resting membrane potential (RMP; Figure 2a) along with significant increase in input resistance (Figure 2b) after treatment with ZD7288. The amount of increase in input resistance was higher at more hyperpolarized voltages, compared to depolarized voltages (Figure 2c), consistent with the HCN channel being a hyperpolarization‐activated ion channel. In addition, treatment with ZD7288 resulted in a significant increase in temporal summation (Figure 2d) and maximum impedance amplitude (Figure 2e), with the increase in maximum impedance amplitude higher at hyperpolarized voltages rather than at depolarized voltages (Figure 2f). We observed a significant increase in action potential firing rate after treatment with ZD7288, especially at intermediate ranges of current values (Figure 2g,h). These physiological changes observed with treatment with ZD7288—hyperpolarization of RMP, increased sub‐ and suprathreshold excitability, enhanced temporal summation, larger changes at hyperpolarized voltages—represent key signatures of effective blockade of HCN channels across different cell types (e.g., Das & Narayanan, 2017; Dembrow et al., 2010; Dickson et al., 2000; Kalmbach et al., 2013; Magee, 1998; Narayanan & Johnston, 2007; Nolan et al., ,^2004^, ^2007^; Pastoll et al., 2020; Poolos et al., 2002). We noted that the impact of blocking HCN channels on individual measurements was heterogeneous, and was observed across all measurements (quantified using multiple measures of degree of variability in Tables 1 and 2). For instance, whereas some cells underwent a small 1 mV hyperpolarization upon HCN channel blockade, others showed a large 5 mV hyperpolarization (Figure 2a).

### 3.2 Blocking barium‐sensitive inward rectifier potassium channels enhanced intrinsic excitability of DG granule cells

**FIGURE 3:**
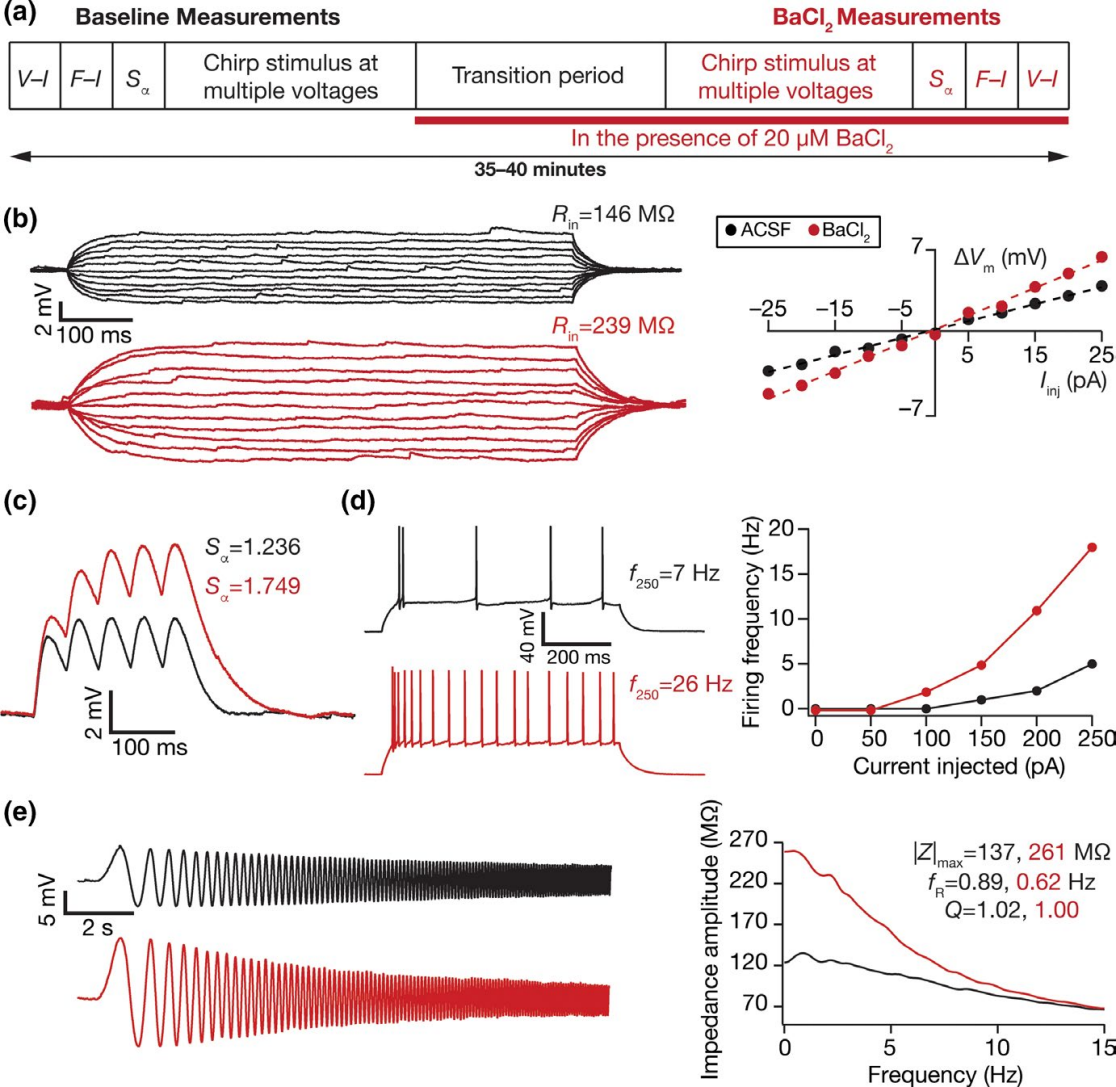
Illustration of the impact of acute treatment with barium chloride (BaCl_2_), an inward rectifier potassium channel blocker, on the electrophysiological properties of DG granule cells. (a) Illustration of the protocol employed for assessing the impact of BaCl_2_ on granule cell physiology. (b) *Left*, Voltage responses of a DG granule cell to 700 ms current pulses of amplitude varying from –25 to +25 pA (in steps of 5 pA), with normal ACSF (*black*) and in the presence of 50 µM BaCl_2_ in the bath (*red*). *Right*, Input resistance (*R*_in_) was calculated as the slope of the *V–I* plot depicting steady‐state voltage response as a function of the injected current amplitude. (c) Voltage response of the example neuron to 5 alpha‐current injections arriving at 20 Hz, depicting temporal summation. Temporal summation ratio (*S*_α_) was computed as the ratio of the amplitude of the fifth response to that of the first. (d) *Left*, Voltage response of the example neuron to a 700 ms current pulse of 250 pA in ACSF (*black*) and in the presence of BaCl_2_ (*red*). *Right*, Frequency of firing plotted as a function of injected current amplitude for the example cell. (e) *Left*, Voltage responses of the example neuron to the *Chirp15* current in ACSF (*black*) and in the presence of BaCl_2_ (*red*). *Right*, Impedance amplitude computed from the *Chirp15* current stimulus and the voltage responses shown on the left. |*Z*|_max_ represents the maximum impedance amplitude, *Q* is resonance strength and resonance frequency is represented by *f*_R_. *V*_RMP_ = −79.1 mV for all traces and measurements depicted here

Barium‐sensitive inward rectifier potassium (K_ir_) channels are known to play a critical role in regulating intrinsic excitability in different neuronal subtypes (Bal & Oertel, 2000; Baruscotti et al., 2010; Borin et al., 2014; Chen & Johnston, 2005; Datunashvili et al., 2018; Day et al., 2005; Dickson et al., 2000; Goldstein et al., 2001; Hibino et al., 2010; Hogg et al., 2001; Kim & Johnston, 2015; Lee & Ishida, 2007; Li et al., 2017; Ma et al., 2003; Malik & Johnston, 2017). Although there are lines of evidence for the expression of K_ir_ channels in DG granule cells (Stegen et al., 2009, 2012; Young et al., 2009), their impact on intrinsic neuronal properties has not been systematically assessed. We tested the impact of treating DG granule cells with 50 µM BaCl_2_, a blocker of K_ir_ channels at lower concentrations (Bal & Oertel, 2000; Baruscotti et al., 2010; Borin et al., 2014; Chen & Johnston, 2005; Datunashvili et al., 2018; Day et al., 2005; Dickson et al., 2000; Goldstein et al., 2001; Hibino et al., 2010; Hogg et al., 2001; Kim & Johnston, 2015; Lee & Ishida, 2007; Li et al., 2017; Ma et al., 2003; Malik & Johnston, 2017; Stegen et al., ,^2009^, ^2012^; Young et al., 2009), by measuring several electrophysiological properties before and after treatment with BaCl_2_ (Figure 3a). We found that treatment with BaCl_2_ enhanced input resistance (Figure 3b), temporal summation (Figure 3c), action potential firing rate (Figure 3d), and impedance amplitude (Figure 3e) of granule cells.

**FIGURE 4:**
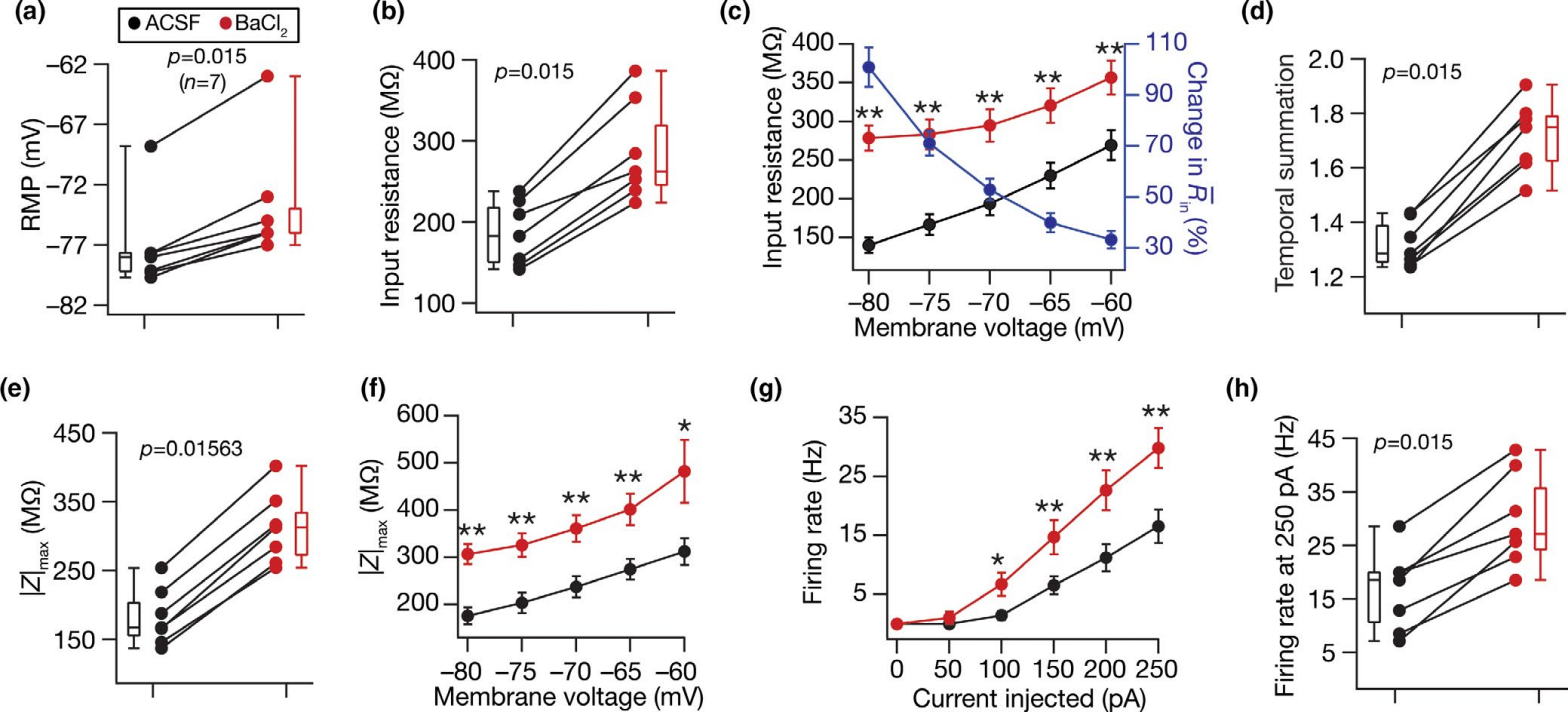
Acute treatment with barium chloride (BaCl_2_), an inward rectifier potassium channel blocker, depolarized resting membrane potential and enhanced sub‐ and suprathreshold excitability of DG granule cells. Population data of measurements from all DG granule cells recorded before and after adding BaCl_2_ to the bath: RMP (a); input resistance, *R*_in_ (b); membrane potential dependence of input resistance (c); temporal summation (d); impedance amplitude, |*Z*|_max_ (e) and its voltage dependence (f); firing rate at 0–250 pA current injection (g) and for 250 pA current injection (h). The Wilcoxon signed rank test was used for *p* value calculation in panels (a–b), (d–e) and (h), for comparing measurements from the same set of cells. For panel (c), (f), and (g) statistical comparisons were performed with paired Student's *t*‐test; *: *p* < 0.05, **: *p* < 0.005

We repeated this protocol across a population of DG granule cells, and found that treatment with BaCl_2_ resulted in significant *depolarization* of RMP (Figure 4a), as opposed to the *hyperpolarization* observed with ZD7288 (Figure 2a). These observations are consistent with the outward versus the inward nature of the current mediated by K_ir_ versus HCN channels, respectively. In addition, BaCl_2_ treatment resulted in significant increases in input resistance (Figure 4b) predominantly at hyperpolarized voltages (Figure 4c), temporal summation (Figure 4d), impedance magnitude (Figure 4e) predominantly at hyperpolarized voltages (Figure 4f), and action potential firing frequency (Figure 4g,h). We noted that the impact of blocking K_ir_ channels on individual measurements was heterogeneous, and was observed across all measurements (Tables 1 and 2). Together, ZD7288 and BaCl_2_ enhanced intrinsic excitability and temporal summation of DG granule cells, pointing to HCN and K_ir_ to be critical resting conductance regulating intrinsic physiology of these neuronal subtypes, with heterogeneous impact on cellular‐scale measurements.

### 3.3 The impact of ZD7288 on intrinsic excitability of DG granule cells persisted even in the presence of barium

**FIGURE 5:**
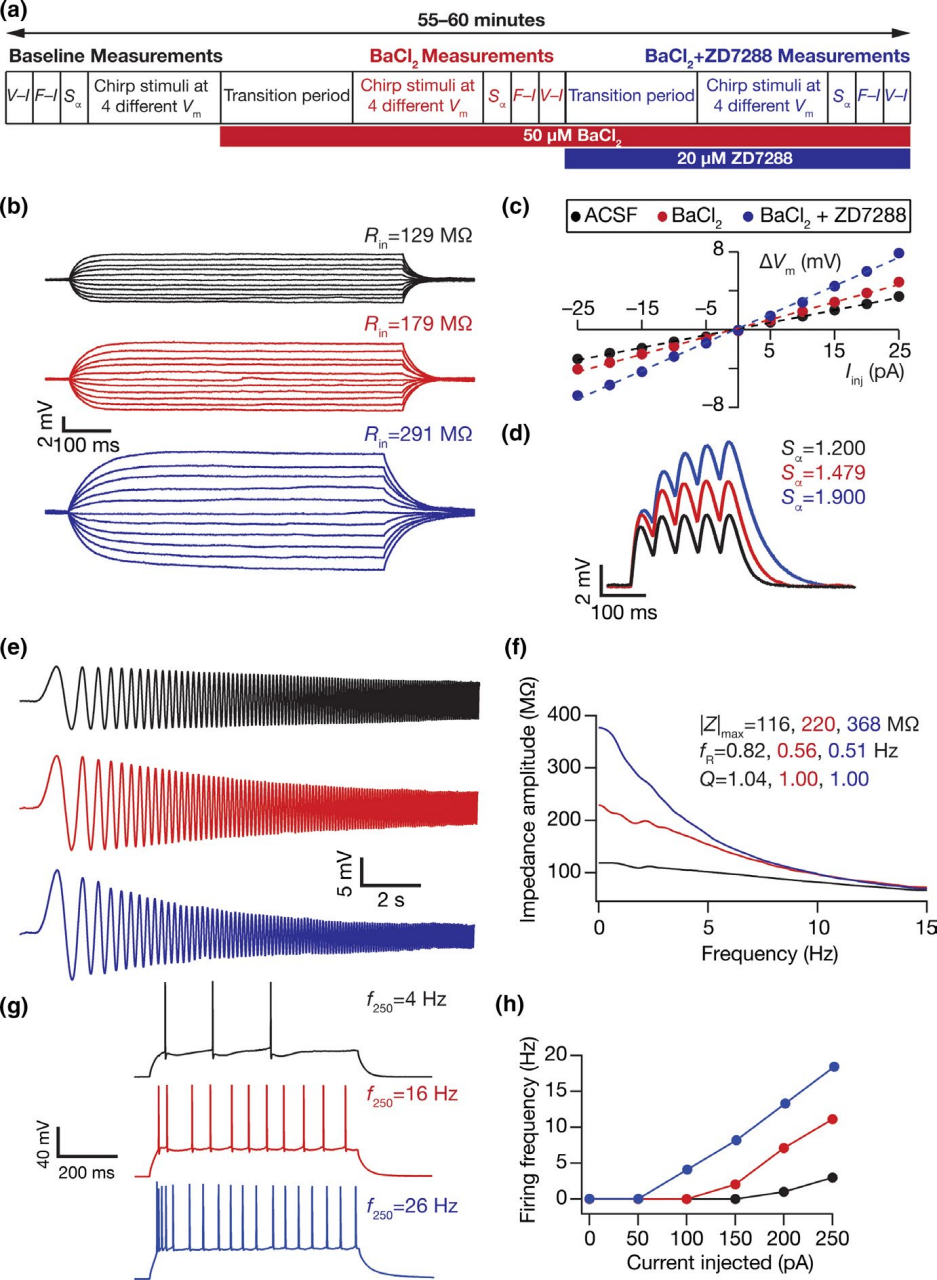
Impact of acute treatment with BaCl_2_ followed by BaCl_2_ + ZD7288 on sub‐ and suprathreshold excitability in an example DG granule cell. (a) Illustration of the protocol employed for assessing the independent impact of BaCl_2_ and ZD7288 on granule cell physiology. (b) Voltage responses of a DG granule cell to 700 ms current pulses of amplitude varying from –25 to +25 pA (in steps of 5 pA), with normal ACSF (*black*) and in the presence of 50 µM BaCl_2_ in the bath (*red*) and in the presence of 50 µM BaCl_2_ and 20 µM ZD7288 in bath (*blue*). (c) Input resistance (*R*_in_) was calculated as the slope of the *V–I* plot depicting steady‐state voltage response as a function of the injected current amplitude. (d) Voltage response of the example neuron to 5 alpha‐current injections arriving at 20 Hz, depicting temporal summation. Temporal summation ratio (*S*_α_) was computed as the ratio of the amplitude of the fifth response to that of the first. (e) Voltage responses of the example neuron to the *Chirp15* current in ACSF (*black*), in the presence of BaCl_2_ (*red*) and in the additional presence of ZD7288 (*blue*). (f) Impedance amplitude computed from the current stimulus shown in Figure 1b (*top*) and the voltage responses shown on the left. (g) Voltage response of the example neuron to a 700 ms current pulse of 250 pA in ACSF (*black*), in the presence of BaCl_2_ (*red*) and in the additional presence of ZD7288 (*blue*). (h) Frequency of firing plotted as a function of injected current amplitude for the example cell. The experiment reported in this figure was performed in the presence of 10 µM CNQX, 10 µM (+) bicuculline, and 10 µM picrotoxin. *V*_RMP_ = −79.2 mV for all traces and measurements depicted here

There are several lines of experimental evidence specifically showing that ZD7288 does not block K_ir_ channels and BaCl_2_ does not block HCN channels, resulting in the use of these pharmacological agents in delineating the individual roles of these resting conductance across several cell types that they co‐express (Bal & Oertel, 2000; Baruscotti et al., 2010; Borin et al., 2014; Datunashvili et al., 2018; Day et al., 2005; Dickson et al., 2000; Hogg et al., 2001; Lee & Ishida, 2007; Li et al., 2017; Ma et al., 2003). To delineate the independent roles of these HCN versus K_ir_ channels on intrinsic properties of DG granule cells, we first measured intrinsic properties in the presence of BaCl_2_, and followed that by measurements in the presence of a mixture of BaCl_2_ and ZD7288 (Figure 5a). Results from an example cell subjected to this protocol showed all measurements—input resistance (Figure 5b), temporal summation (Figure 5c), impedance amplitude (Figure 5e,f), and action potential firing rate (Figure 5g,h)—to increase with BaCl_2_ treatment (consistent with Figures 3 and 4), and to show further enhancement upon joint treatment with BaCl_2_ and ZD7288.

**FIGURE 6:**
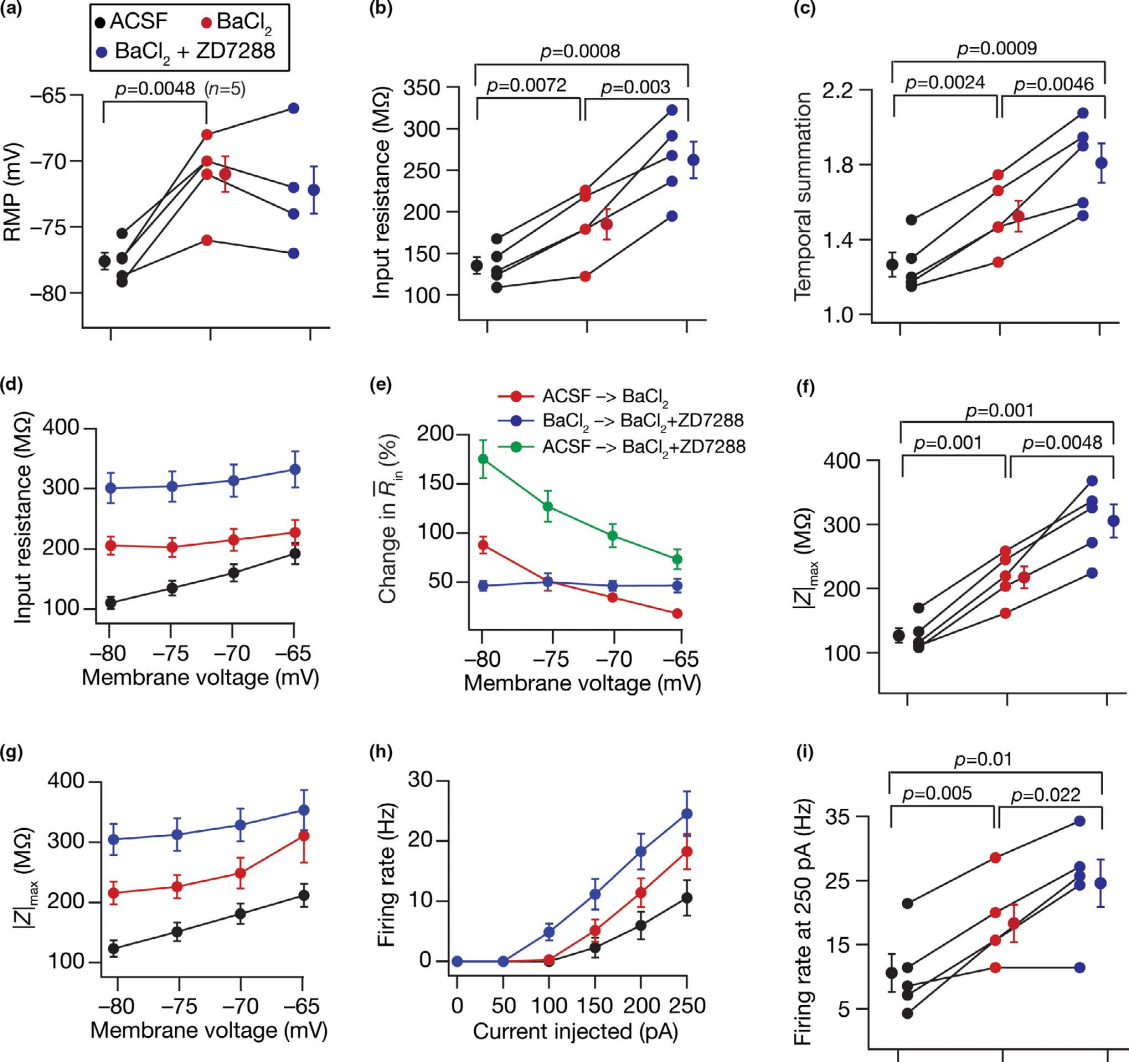
ZD7288 enhanced sub‐ and suprathreshold excitability of DG granule cells beyond the excitability enhancement induced by BaCl_2_ treatment. (a–i) Population data of measurements from all DG granule cells recorded in ACSF, then in presence of 50 µM BaCl_2_ (red) and in the presence of 50 µM BaCl_2_ and 20 µM ZD7288 in the bath (blue): RMP (a); input resistance, *R*_in_ (b); temporal summation (c); membrane potential dependence of input resistance (d); percentage change in input resistance as a function of membrane potential (e): impedance amplitude, |*Z*|_max_ (f) and its voltage dependence (g); firing rate for 0–250 pA current injection (h) and for 250 pA current injection (i). For all panels statistical comparisons were performed with paired Student's *t*‐test; *: *p* < 0.05, **: *p* < 0.005. For panels (d) and (g), across‐group measurements were significantly different (*p* < 0.05) from each other for all measured voltages. For panel (h), across‐group measurements of action potential firing rates were significantly different (*p* < 0.05) for current injections in the range 150–250 pA; for 100‐pA current injection, firing rate in the (BaCl_2_ + ZD7288) group was significantly different (*p* < 0.05) from the other two groups. All experiments reported in this figure were performed in the presence of 10 µM CNQX, 10 µM (+) bicuculline, and 10 µM picrotoxin

To confirm these observations that ZD7288 enhanced intrinsic excitability and temporal summation independent of the enhancement induced by BaCl_2_, we repeated this experiment (Figure 5a) on a population of granule cells. Whereas BaCl_2_ treatment resulted in significant depolarization of RMP (Figure 6a), consistent with our earlier observations (Figure 4a), the subsequent joint treatment with BaCl_2_ and ZD7288 resulted in hyperpolarization of RMP in the same set of neurons. Importantly, there were significant increases in input resistance across voltages and temporal summation upon treatment with BaCl_2_, which showed further significant increases in the joint presence of BaCl_2_ and ZD7288 (Figure 5c–e). Maximal impedance amplitude and action potential firing rate also followed similar trends (Figure 5f–i), confirming that HCN‐channel blockade has a significant impact on DG granule cell excitability and resting properties, which was independent of K_ir_ channels. We also noted that the impact of blocking HCN or K_ir_ channels on individual measurements was heterogeneous, with certain measurements in some cells showing a relatively larger dependence on HCN channels while other cells showed a larger dependence on K_ir_ channels for the same measurement. Together, our experiments with ZD7288 and BaCl_2_ individually (Figures 1–2 and 3–4, respectively) and together (Figures 5 and 6) demonstrated that blockade of either HCN or barium‐sensitive K_ir_ channels independently yield significant increases in sub‐ and suprathreshold excitability of DG granule cells.

**FIGURE 7:**
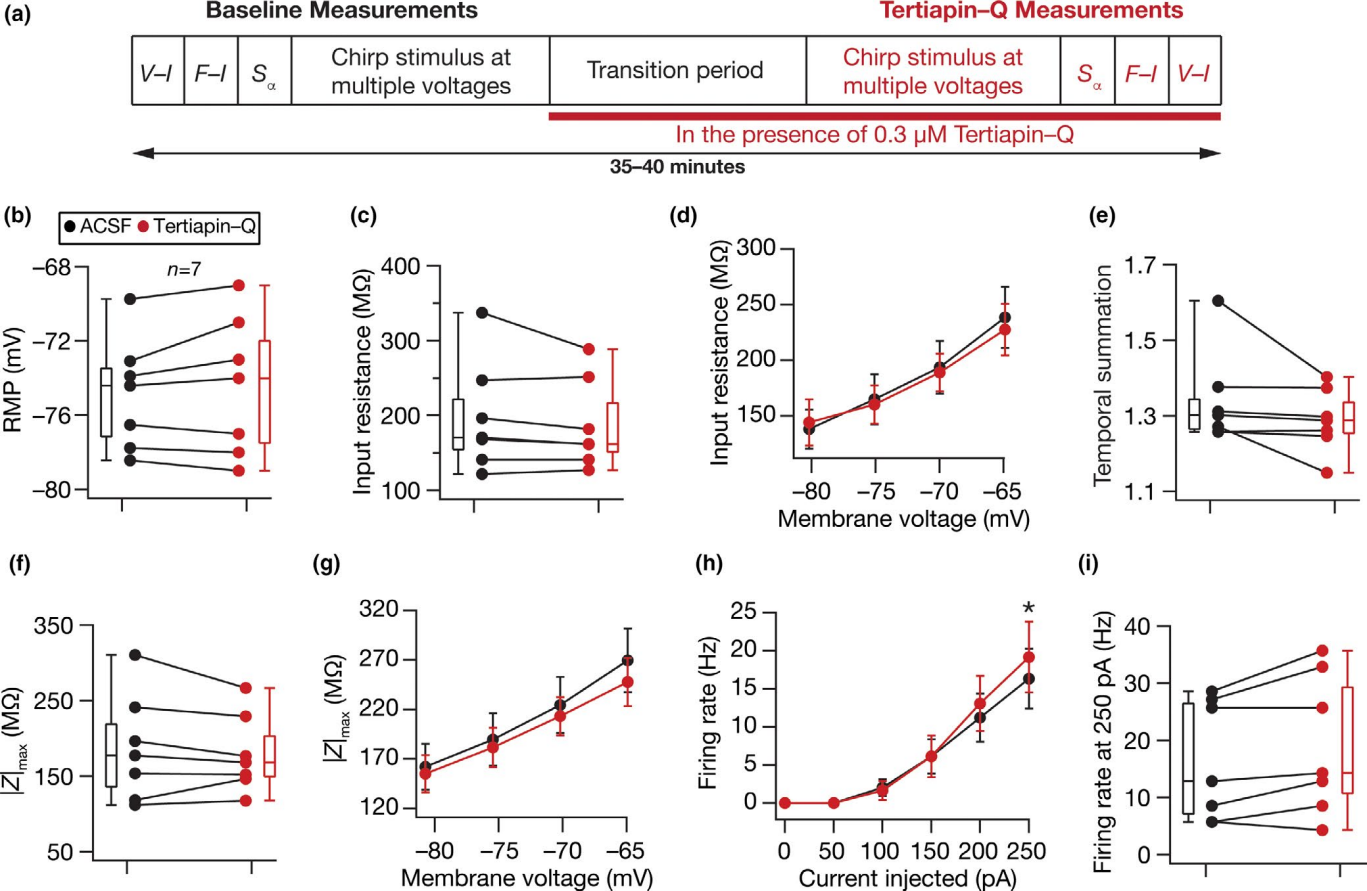
Acute treatment with tertiapin‐Q, a blocker of specific subtypes of inward rectifier potassium channels, yielded no significant change in physiological measurements of DG granule cells. (a) Illustration of the protocol employed for assessing the impact of tertiapin‐Q on granule cell physiology. (b–i) Population data of measurements from all DG granule cells recorded before (*black*) and after adding 0.3 µM tertiapin‐Q (*red*) to the bath: RMP (b); input resistance, *R*_in_ (c); membrane potential dependence of input resistance (d); temporal summation (e); impedance amplitude, |*Z*|_max_ (f) and its voltage dependence (g); firing rate at 0–250 pA current injection (h) and for 250 pA current injection (i). The Wilcoxon signed rank test was used for *p* value calculation in panels (b–c), (e–f), and (i), for comparing measurements from the same set of cells. For panel (d), (g), and (h) statistical comparisons were performed with paired Student's *t*‐test; *: *p* < 0.05. All experiments reported in this figure were performed in the presence of 10 µM CNQX, 10 µM (+) bicuculline and 10 µM picrotoxin

Tertiapin Q is a blocker of specific subtypes of inward rectifier potassium channels, which have been shown to play important roles in governing excitability of different cell types (Chen & Johnston, 2005; Jin & Lu, 1999; Kim & Johnston, 2015; Malik & Johnston, 2017; Zhang et al., 2013). We measured intrinsic physiological properties of DG granule cells before and after treatment with 0.3 µM tertiapin‐Q (Figure 7a). We found none of the measured physiological measurements to be significantly different before versus after treatment of tertiapin‐Q (Figure 7, Tables 1 and 2), together pointing to the dominance of barium‐sensitive inward rectifying potassium channels (Figures 3 and 6) over those sensitive to tertiapin‐Q in DG granule cells.

### 3.4 Treatment with riluzole, a blocker of persistent sodium channels, reduced intrinsic excitability of DG granule cells

**FIGURE 8:**
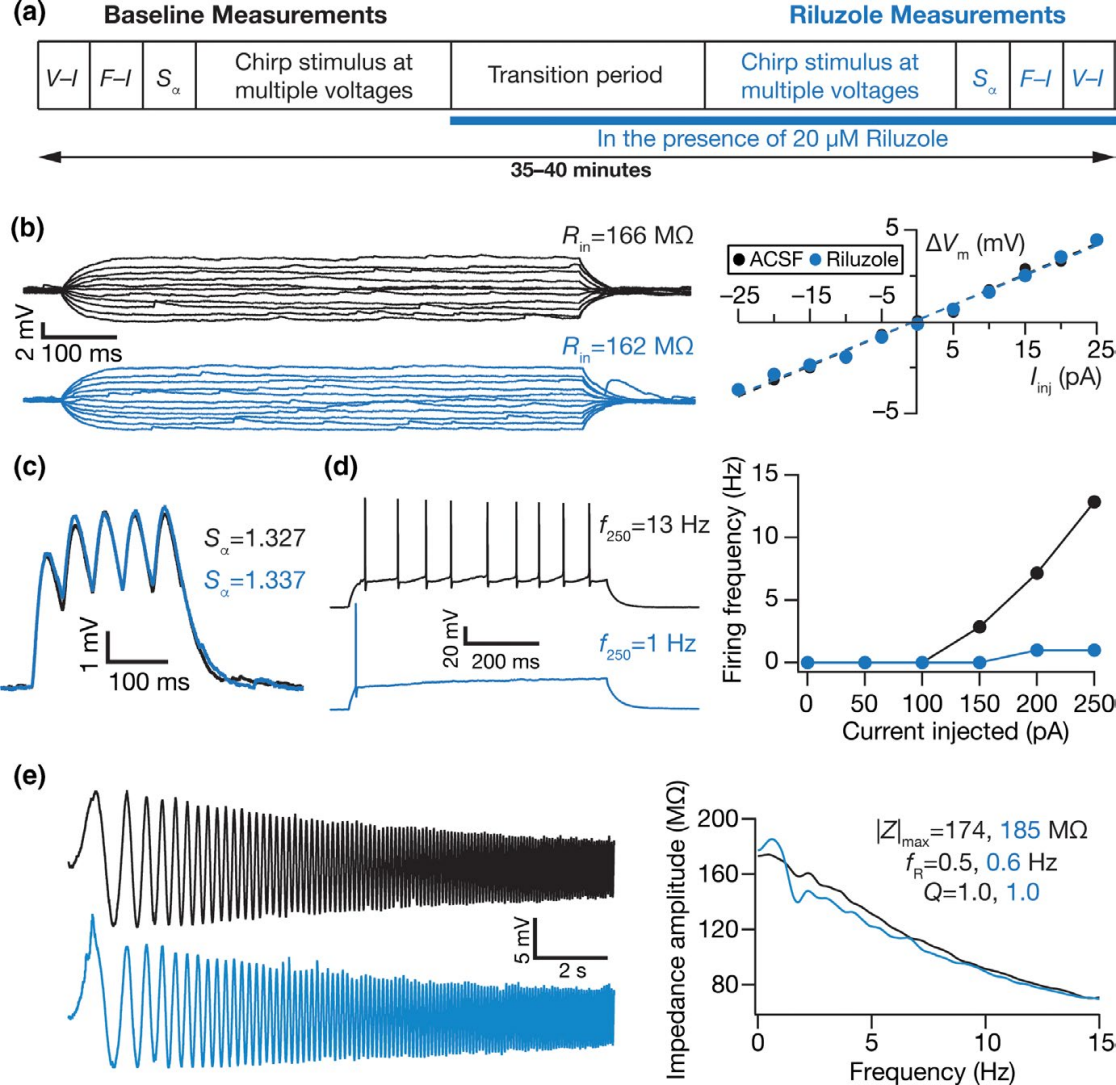
Illustration of the impact of acute treatment with riluzole, a persistent sodium channel blocker, on several subthreshold and action potential measurements of DG granule cells. (a) Illustration of the protocol employed for assessing the impact of riluzole on granule cell physiology. (b) *Left*, Voltage responses of a DG granule cell to 700 ms current pulses of amplitude varying from –25 to +25 pA (in steps of 5 pA), with normal ACSF (*black*) and in the presence of riluzole in the bath (*blue*). *Right*, Input resistance (*R*_in_) was calculated as the slope of the *V–I* plot depicting steady‐state voltage response as a function of the injected current amplitude. (c) Voltage response of the example neuron to 5 alpha‐current injections arriving at 20 Hz, depicting temporal summation. Temporal summation ratio (*S*_α_) was computed as the ratio of the amplitude of the fifth response to that of the first. (d) *Left*, Voltage response of the example neuron to a 700 ms current pulse of 250 pA in ACSF (*black*) and in the presence of riluzole (*blue*). *Right*, Frequency of firing plotted as a function of injected current amplitude for the example cell. (e) *Left*, Voltage responses of the example neuron to the *Chirp15* current in ACSF (*black*) and in the presence of Riluzole (*blue*). *Right*, Impedance amplitude computed from the current stimulus shown in Figure 1b (*top*) and the voltage responses shown on the left. *V*_RMP_ = −72.6 mV for all traces and measurements depicted here

**FIGURE 9:**
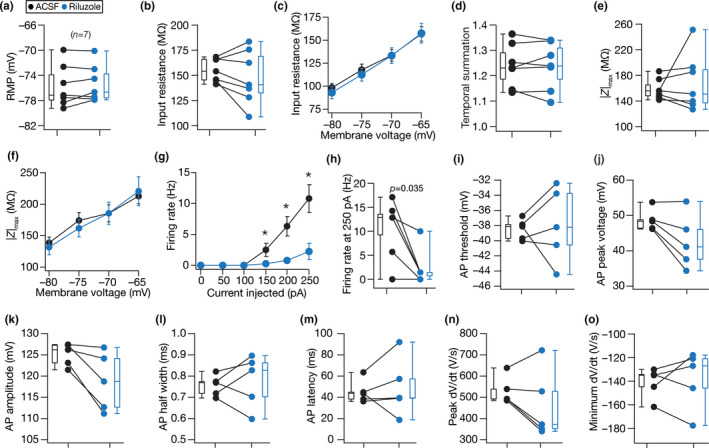
Acute treatment with riluzole, a persistent sodium channel blocker, reduced the firing rate of DG granule cells without altering their subthreshold physiological characteristics. Population data of measurements from all DG granule cells recorded before (black) and after (blue) adding riluzole to the bath: RMP (a); input resistance, *R*_in_ (b); membrane potential dependence of input resistance (c); temporal summation (d); impedance amplitude, |*Z*|_max_ (e) and its voltage dependence (f); firing rate at 0–250 pA current injection (g) and for 250 pA current injection (h); AP threshold (i); AP peak voltage (j); AP amplitude (k); AP half‐width (l); latency to first spike (m); peak (n) and minimum (o) dV/dt of the action potential waveform. Although there was a trend of consistent reduction in AP amplitude and AP peak voltage, none of the measurements depicted in this figure were statistically significant with Wilcoxon signed rank test. For panel (g) statistical comparisons were performed with paired Student's *t*‐test; *: *p* < 0.05

Sodium channels mediating persistent currents are critically involved in regulating intrinsic excitability in several classes of neurons (Crill, 1996; Das & Narayanan, 2015; Epsztein et al., 2010; Fransen et al., 2004; Gutfreund et al., 1995; Hsu et al., 2018; Hu et al., 2002; Su et al., 2001; Vervaeke et al., 2006). Motivated by the expression of a persistent sodium (NaP) channel in DG granule cells predominantly in axon initial segments, and the role of these channels in regulating AP firing properties (Artinian et al., 2011; Beining et al., 2017; Crill, 1996; Ellerkmann et al., 2003; Epsztein et al., 2010; Kress et al., 2010), we first assessed a role for NaP channel in regulating DG granule cell excitability. We performed whole‐cell current‐clamp recordings, measured baseline sub‐ and suprathreshold properties of DG granule cells, and then repeated these measurements in the presence of 20 µM riluzole (Figure 8a), an established inhibitor of persistent sodium currents (Song et al., 1997; Urbani & Belluzzi, 2000). We found that riluzole introduced no significant changes to RMP, subthreshold excitability across different voltages or *S*_α_ (Figures 8 and 9). In contrast, there was a pronounced reduction in firing rate of granule cells after application of riluzole (Figure 8c,d, Figure 9g,h), without significant changes to AP properties (Figure 9i–o). These results provided evidence for a role for riluzole‐sensitive persistent sodium channels in the regulation of suprathreshold excitability in DG granule cells. In addition, we noted that the impact of blocking NaP channels on individual measurements was heterogeneous, and was observed across all measurements (Tables 1 and 2). As examples, this heterogeneity is clear with reference to input resistance (Figure 9b) and maximal impedance amplitude (Figure 9e), where some cells showed an increase whereas others showed a decrease upon treatment with riluzole, potentially owing to synergistic interactions with other channels expressed in those neurons.

### 3.5 Granule cells manifested the twin signatures for the expression of ion‐channel degeneracy: Many‐to‐many mapping and heterogeneous impact of ion‐channel blockade

**FIGURE 10:**
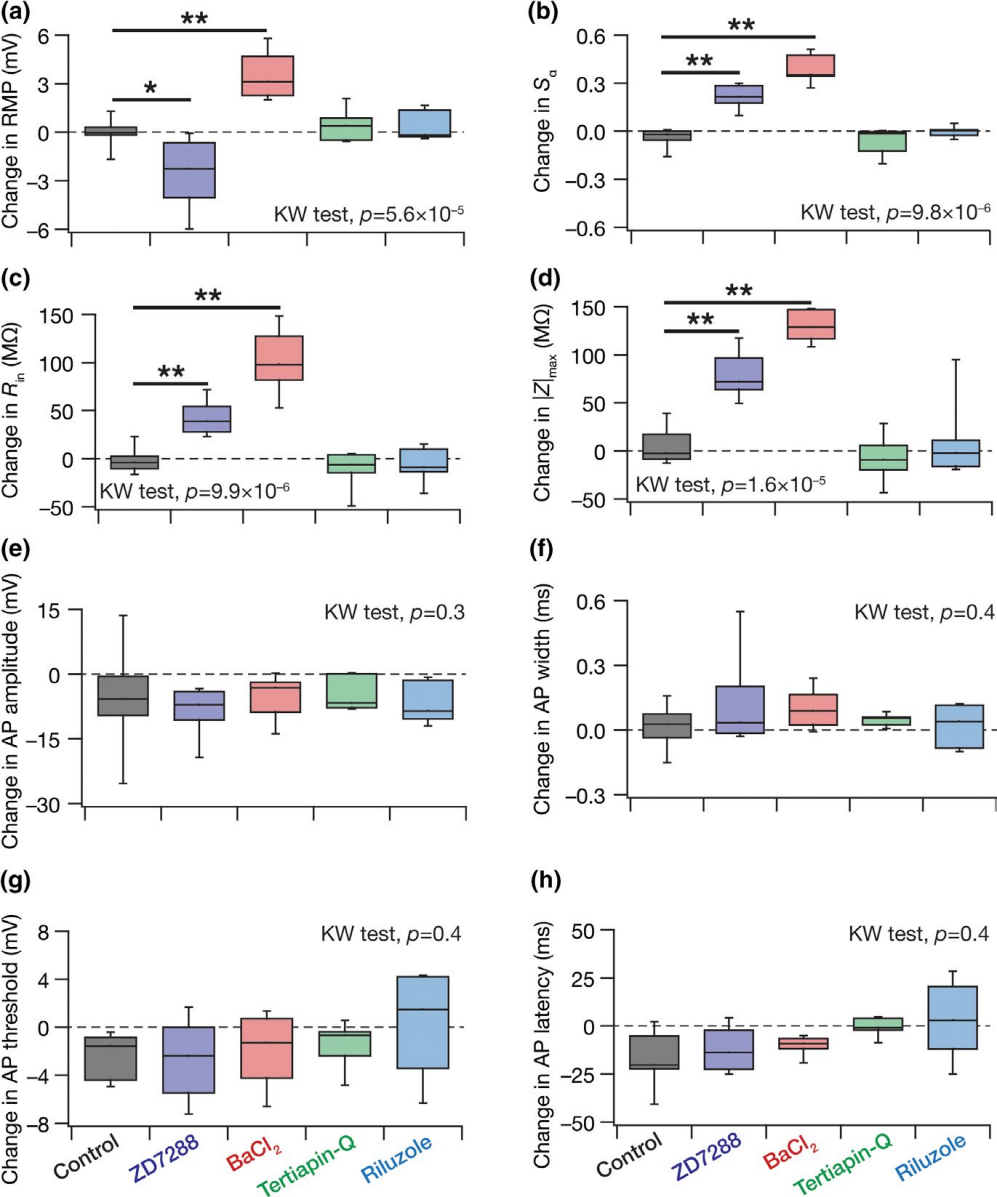
Quantum of changes in different sub‐ and suprathreshold measurements in response to individual ion‐channel blockade provide experimental evidence for the twin signatures for the expression of ion‐channel degeneracy. (a–h) Changes in resting membrane potential (a), temporal summation ratio (b), input resistance (c), maximum impedance amplitude (d), action potential amplitude (e), action potential width (f), action potential threshold (g), and action potential latency (h). Presented are data from the time‐matched control group (Control), and pharmacological agent‐induced changes with reference to ZD7288, BaCl_2_, tertiapin Q, and riluzole. For data associated with pharmacological agents, shown are the differences between the respective measurements after and before treatment with the agent (*i*.*e*., after–before). All data are presented as quartiles. Control, *n* = 10; ZD7288, *n* = 8; BaCl_2_, *n* = 7; tertiapin Q, *n* = 7; riluzole, *n* = 7. KW test: Kruskal–Wallis test across all groups. *p < 0.05; **p < 0.005: Wilcox rank sum test with reference to the “Control” group. Percentage changes in all sub‐ and suprathreshold changes, and *p* values corresponding to KW and Wilcox rank sum test are presented in Tables 1 and 2

Ion‐channel degeneracy is the ability of disparate ion‐channel combinations to yield characteristic cellular‐scale physiological measurements. Direct experimental verification of the expression of ion‐channel degeneracy requires the assessment of the contributions of *all* ion channels to electrophysiological measurements *in the same neuron*, and comparing these contributions across different neurons showing characteristic physiological signatures. This entails electrophysiological measurements involving sequential application and washing of different pharmacological agents targeting each of the several ion channels expressed in neurons. However, owing to time limitations of electrophysiology experiments and properties of pharmacological agents (e.g., difficulty in washing pharmacological agents, such as ZD7288, after application; specificity of pharmacological agents), this is currently infeasible. Thus, in this study, we sought experimental evidence for the manifestation of two signature characteristics, derived from prior computational models, that point to the expression of ion‐channel degeneracy. Our experiments and analyses involving different pharmacological agents demonstrated strong lines of evidence for both these characteristic signatures (Figure 10):

1. **Many‐to‐many mapping:** We first noted that the same electrophysiological measurement was affected by different ion channel blockers (Figure 10; Tables 1 and 2). For instance, resting membrane potential (RMP; Figure 10a) was depolarized with the application of BaCl_2_, but hyperpolarized with the application of ZD7288. This constitutes an example of *many‐to‐one mapping* between ion channels (HCN and K_ir_ in this example) and individual physiological measurements (RMP). Second, we observed that blocking any given ion channel altered several measurements. For instance, application of ZD7288 hyperpolarized RMP (Figure 10a), increased temporal summation (Figure 10b), and enhanced input resistance (Figure 10c) and maximal impedance amplitude (Figure 10d). This provides an illustration for the *one‐to‐many mapping* between a specific ion channel (HCN in this example) and physiological measurements (RMP, $R_{\text{in}}$, *S*_α_, ${\left|Z\right|}_{\text{max}}$ in this case). Together, the 21 measurements and the four different ion‐channel blockers employed here (Figures 1, 2, 3, 4, 5, 6, 7, 8, 9, 10; Tables 1 and 2) present several lines of evidence for many‐to‐many, but *not* all‐to‐all, mappings between ion channels and physiological measurements. These provide electrophysiological evidence for the first signature characteristic for the expression of ion‐channel degeneracy that we sought to identify.
2. **Heterogeneity in changes introduced by blocking individual ion channels:** We assessed the range of changes observed in individual measurements with any of the four ion‐channel blockers. Across measurements, we noted that the changes spanned a wide range and were not clustered around a specific mean/median value. For instance, with the application of ZD7288, RMP changed between ~0 mV (no change) and 6 mV hyperpolarization (Figure 10a) across different cells. Another example was with the application of BaCl_2_, which changed input resistance by 50 MΩ in certain cells to 150 MΩ in others (Figure 10c). Quantitatively, there was considerable heterogeneity in the changes in each measurement with the application of individual pharmacological agents, as assessed through three standard measures of variability (standard deviation, interquartile range, and coefficient of variation; Tables 1 and 2). These experimental data provide electrophysiological evidence for the second signature characteristic for the expression of ion‐channel degeneracy that we sought to identify.

Together, electrophysiological data spanning several resting, sub‐ and suprathreshold measurements—involving four different pharmacological agents targeting prominent non‐inactivating, subthreshold‐activated ion channels—provided evidence for the manifestation of the two electrophysiological signatures for the expression of ion‐channel degeneracy in DG granule cells.

## 4 DISCUSSION

The principal goal of our study was to seek electrophysiological evidence for two signature characteristics that point to the expression of ion‐channel degeneracy in the emergence of single‐neuron physiology of DG granule neurons, which has been predicted by computational studies (Beining et al., 2017; Mishra & Narayanan, 2019, 2021). Specifically, we reasoned that the expression of ion‐channel degeneracy would translate to the ability of multiple ion channels to alter the same functional measurement and would also result in heterogeneous impact on the same measurement in different cells. In testing our postulate on ion‐channel degeneracy, here we provide direct electrophysiological evidence that several resting and subthreshold‐activated ion channel conductance heterogeneously regulate intrinsic neuronal physiology of DG granule cells (Figure 10). We arrived at this conclusion by employing an array of physiological measurements, which were measured before and after application of pharmacological agents that targeted specific ion channels. The direction of pharmacology‐induced changes in the several physiological measurements, in conjunction with insights from the literature about the role of specific ion channels on these measurements, provided clear lines of evidence on the lack of one‐to‐one relationships between ion channel subtypes and the different physiological measurements. Our findings provide experimental evidence for the expression of ion‐channel degeneracy in DG granule cells, and have important implications for robustness of these neurons and their networks in the face of external perturbations and circuit heterogeneities.

### 4.1 Many‐to‐many mapping between ion channels and neuronal intrinsic properties point to ion‐channel degeneracy in DG granule cell physiology

Our findings demonstrate that multiple ion channels regulate each of the several physiological measurements of granule cells (Figure 10). For instance, we provide evidence that neuronal HCN, K_ir,_ and NaP channels regulated firing rate, whereas input resistance was altered by blockade of HCN and K_ir_ channels but not by blocking NaP channels. These point to a many‐to‐many, but not all‐to‐all (that is, blocking no individual ion channel affected *all* the measurements), relationship between ion channels and physiological measurements. Such a many‐to‐many regulatory relationship is central to the expression of degeneracy, defined as the ability of disparate structural components in achieving similar functional outcomes (Edelman & Gally, 2001). In this case, if intrinsic excitability or temporal summation was considered as the specific function under consideration, our results show that disparate ion channels or their combinations could achieve similar excitability or summation. This form of degeneracy points to the emergence of similar cellular‐scale function (signature neuronal intrinsic properties) through disparate combinations of structural components in the molecular scale (different ion channel subtypes). The ability of disparate ion‐channel combinations to elicit cell type‐specific characteristic cellular‐scale physiological signatures (including intrinsic excitability) provides an advantage by tremendously increasing the degrees of freedom available to a neuron to achieve robustness in functionality (Goaillard & Marder, 2021). Such ion‐channel degeneracy also provides a clear explanation for why different neurons with similar signature cellular‐scale function exhibit widespread heterogeneity in their ion‐channel composition, as this would be a direct consequence of different sets of ion channels mediating cellular functions in different neurons (Goaillard & Marder, 2021; Goaillard et al., 2009; Golowasch et al., 1992; Ma & Koester, 1996; Rathour et al., 2016; Swensen & Bean, 2003, 2005).

Ion channel degeneracy plays a critical role in achieving robust function despite variability in ion channel conductances expressed in different neurons of the same subtype, because of the several degree of freedom available to the neuron through which signature functional outcomes could be achieved (Anirudhan & Narayanan, 2015; Basak & Narayanan, 2018, 2020; Beining et al., 2017; Das & Narayanan, 2015; Das et al., 2017; Drion et al., 2015; Jain & Narayanan, 2020; Mishra & Narayanan, 2019; Mittal & Narayanan, 2018; Mukunda & Narayanan, 2017; O'Leary, 2018; Onasch & Gjorgjieva, 2020; Rathour et al., 2016; Rathour & Narayanan, 2012a, 2014, 2019; Seenivasan & Narayanan, 2020). Such ion‐channel degeneracy also provides an explanation for the heterogeneities observed in the impact of specific channel blockade on different cells of the same subtype. For instance, the impact of blocking HCN channels on input resistance is higher than that of blocking K_ir_ channels in certain neurons, whereas the scenario is flipped for certain other neurons (Figure 6b). These observations point to the dominance of different ion channels in regulating specific functions in different neurons showing similar functional properties. Although our study focused on four subtypes of ion channels, future studies could extend such analyses to other ion channels, including calcium‐activated potassium channels, voltage‐gated calcium channels, and other sodium and potassium channels in DG granule cells. Based on earlier computational predictions in DG granule cells (Beining et al., 2017; Mishra & Narayanan, 2019, 2021), and on electrophysiological observations here, we postulate that the incorporation of additional channels to the analyses would further increase the degrees of freedom available to the neuron in maintaining similar function.

Together, our results emphasize ion‐channel degeneracy and the associated heterogeneous impact of different ion channel subtypes on neuronal functions to constitute defining characteristics of neuronal physiology. Our results demonstrate the impact of multiple ion channels on the same set of physiological measurements, and emphasize the need to account for ion‐channel degeneracy in interpreting physiological experiments and in understanding the etiology of or designing remedies for pathological conditions (Edelman & Gally, 2001; Goaillard & Marder, 2021; Goaillard et al., 2009; Leonardo, 2005; Marder, 2011; Marder & Goaillard, 2006; Marder & Taylor, 2011; Price & Friston, 2002; Rathour & Narayanan, 2019; Ratté et al., 2014).

### 4.2 HCN channels and neuronal intrinsic measurements

The expression of HCN channels results in a depolarization of RMP, consequent to HCN channels mediating a resting inward (depolarizing) current. Consequently, blockade of HCN channels results in the hyperpolarization of RMP, consistent with our observations (Figure 2a). However, if HCN channels mediate an *inward* current, why does their blockade result in an enhancement of intrinsic excitability and temporal summation (Figure 2b–h)? Should not the expression of an inward current result in *enhanced* intrinsic excitability? Should not the blockade of such an inward current lead to a *reduction* in intrinsic excitability? Although HCN channels mediate inward currents, there are important characteristics associated with them that allow them to behave as a *restorative conductance* that suppresses neural excitability.

First, under resting conditions, the current through open HCN channels yields an inward current depolarizing the RMP. However, the open HCN channels that yield this current contribute to an enhanced resting conductance, which translates to a reduction in input resistance thereby suppressing intrinsic excitability. Thus, the impact of the inward current is to depolarize the membrane toward action potential threshold (potentially increasing excitability), whereas the impact of the associated enhanced conductance is to suppress excitability. This conductance‐current balance (Mishra & Narayanan, 2015) consequent to the expression of HCN channels has been studied under different scenarios (Breton & Stuart, 2009; Chen et al., 2001; Dyhrfjeld‐Johnsen et al., 2009; Fan et al., 2005; Gasparini & DiFrancesco, 1997; George et al., 2009; He et al., 2014; Hutcheon & Yarom, 2000; Kim et al., 2012; Lippert & Booth, 2009; Magee, 1998; Migliore & Migliore, 2012; Mishra & Narayanan, 2015; Narayanan & Johnston, 2007, 2008; Noam et al., 2011; Pape, 1996; Pavlov et al., 2011; Robinson & Siegelbaum, 2003; Rosenkranz & Johnston, 2006; Santoro & Baram, 2003; Shah, 2014), and is one of the principal reasons for the complexities associated with analyzing the impact of their expression on neural excitability.

Second, and more importantly, the gating properties of HCN channels allow them to act as *restorative* conductances when they respond to externally driven *changes to membrane potential*. Specifically, although HCN channels mediate an inward current, they mediate *hyperpolarization‐activated* inward currents, which allow them to actively *suppress* any voltage deflection (Hutcheon & Yarom, 2000). To illustrate this, consider an externally driven hyperpolarization introduced by synaptic inputs or current injection. This hyperpolarization would *activate* HCN channels resulting in an additional inward current, which by definition depolarizes the membrane. Similarly, an external depolarization would *deactivate* HCN channels, turning off an inward current and eliciting a hyperpolarization. Thus, an externally driven hyperpolarization *depolarizes* the membrane, whereas a depolarization *hyperpolarizes* the membrane, together implying *bidirectional suppression* of any externally driven voltage deflection by HCN channels. This *restorative* property of HCN channels, directly consequent to their voltage‐dependent gating profile, constitutes an important reason for why blockade of HCN channels enhance input resistance and firing rate across different cell types (Gasparini & DiFrancesco, 1997; Magee, 1998). Whereas the balance between resting current and resting conductance provides a *static* (resting) picture on conductance‐current balance, the restorative property emerges as a consequence of the *dynamics* associated with voltage evolution in response to external changes in membrane voltage. Interestingly, this restorative property of HCN channels (i.e., the ability to suppress voltage deflections), in conjunction with their slow activation/deactivation kinetics, allows them to act as a resonating conductance (Hutcheon & Yarom, 2000). Resonating conductances predominantly suppress low‐frequency voltage deflections and mediate frequency selectivity, in addition to suppressing temporal summation in neurons (Das et al., 2017; Hutcheon & Yarom, 2000; Magee, 1998; Narayanan & Johnston, 2007, 2008).

Finally, the impact of HCN channels on neural excitability and other intrinsic properties also heavily relies on the other ion channels that are expressed in the neuron. In general, neuronal physiology emerges from synergistic interactions between different ion channels expressed in the neuron. The impact of all ion channels on neuronal function is not a linear sum of the impact of individual ion channels when they are independently present, but consequent to strong voltage‐ and calcium‐driven interactions across different ion channels. A simple illustration of this is an action potential, where voltage‐driven interactions between sodium *and* delayed rectifier potassium channels synergistically yield action potentials (Hodgkin & Huxley, 1952). With specific reference to HCN channels, the impact of the co‐expression of other ion channels (including ionotropic receptors) on neuronal physiology with HCN channels have been analyzed with reference to different functional measurements in different neuronal subtypes. Some examples of such analyses involving the impact of HCN channel interactions with other ion channels include: sub‐ and suprathreshold measurements including neuronal resonance (Kelley et al., 2021; Migliore & Migliore, 2012; Mishra & Narayanan, 2015; Narayanan & Johnston, 2008; Rathour & Narayanan, 2012a, 2012b, 2014) , synaptic response dynamics (George et al., 2009; Kelley et al., 2021; Mishra & Narayanan, 2015), short‐ (Huang et al., 2011; Mukunda & Narayanan, 2017) and long‐term (Anirudhan & Narayanan, 2015; Honnuraiah & Narayanan, 2013; Narayanan & Johnston, 2010) plasticity profiles, spike‐triggered average and coincidence detection (Das & Narayanan, 2014, 2015, 2017; Das et al., 2017; Jain & Narayanan, 2020; Khurana et al., 2011, 2012; Mathews et al., 2010; Ratte et al., 2013), local field potentials (Ness et al., 2018; Reimann et al., 2013; Sinha & Narayanan, 2015), and the impact of gliotransmission on hippocampal dendrites (Ashhad & Narayanan, 2016).

In summary, the resting conductance‐current balance, the restorative dynamics of HCN channels consequent to their gating properties, and quantitative interactions of HCN channels with other ion channels, together synergistically contribute to the specific impact of HCN channels on any physiological measurement (including neuronal excitability). The enhancement of intrinsic excitability and temporal summation (Figure 2) consequent to HCN channel blockade is consistent with the gating properties of HCN channels and their impact on neuronal physiology.

### 4.3 Absence of membrane potential resonance and voltage sag in DG granule cells

Although we present evidence that DG granule cells are endowed with HCN channels (Figures 1 and 2, Figures 5 and 6), these neurons did not express prominent voltage sag or membrane potential resonance, electrophysiological signatures that are typically associated with the expression of HCN channels (Das & Narayanan, 2014, 2017; Dhupia et al., 2014; Engel et al., 2008; Erchova et al., 2004; Gimbarzevsky et al., 1984; Giocomo & Hasselmo, 2009; Gutfreund et al., 1995; Haas & White, 2002; Hu et al., ,^2002^, ^2009^; Hutcheon & Yarom, 2000; Narayanan & Johnston, 2007; Pike et al., 2000; Rathour et al., 2016; Rathour & Narayanan, 2012a). The absence of voltage sag and membrane potential resonance persisted across all neurons with any of the different pharmacological treatments applied. In this context, it should be noted that while the presence of sag or resonance reflects the expression of a resonating conductance (such as HCN channels), the absence of sag and resonance does not imply the absence of resonating conductances. Theoretically, the expression of sag and resonance is emergent based on synergistic interactions among several neuronal components, including the passive properties of the cell, the activation/inactivation time constant of the resonating conductance and the expression of other channels (Das et al., 2017; Hutcheon et al., 1996a, 1996b; Hutcheon et al., 1994; Hutcheon & Yarom, 2000; Narayanan & Johnston, 2007, 2008; Rathour et al., 2016; Rathour & Narayanan, 2012a, 2019). There is experimental evidence that HCN channels in DG granule cells are endowed with extremely slow kinetics (Surges et al., 2012), with the activation time constant in the ~500 ms range. The well‐established inverse relationship between the activation time constant of the resonating conductance and the expression of resonance frequency or voltage sag (Hutcheon et al., 1996a; Hutcheon & Yarom, 2000; Narayanan & Johnston, 2008; Rathour & Narayanan, 2012a) is a possible explanation for the lack of these signature electrophysiological characteristics (Mishra & Narayanan, 2020). Future studies should perform systematic cell‐attached recordings along the axo–somato–dendritic axis of DG granule cells to characterize HCN and other ion channels expressed therein, and quantitatively address the question on membrane potential resonance with computational approaches.

### 4.4 Future directions

Given the well‐established heterogeneities and gradients in ion‐channel expression profiles, the results reported here should be considered specific for the somata of mature granule cells in the crest sector of the middle hippocampus in the dentate gyrus of adult male Sprague–Dawley rats. As ion channel expression and intrinsic properties has been shown to vary tremendously in other neuronal subtypes along different anatomical axes (Arnold et al., 2019; Cembrowski et al., 2016; Cembrowski & Spruston, 2019; Danielson et al., 2016; Dougherty et al., 2012, 2013; Giocomo & Hasselmo, 2009; Giocomo et al., 2007; Hönigsperger et al., 2015; Kjelstrup et al., 2008; Lee et al., 2014; Malik et al., 2016; Malik & Johnston, 2017; Marcelin et al., 2012; Maroso et al., 2016; Mizuseki et al., 2011; Pastoll et al., 2020; Soltesz & Losonczy, 2018; Strange et al., 2014; Valero et al., 2015), future studies should explore the ionic basis of intrinsic physiology of granule cells across somato–dendritic, infrapyramidal–suprapyramidal, dorso–ventral, and superficial–deep axes of the dentate gyrus. It is essential to critically assess gradients in the biophysical, morphological, and physiological properties of granule cells across these different axes, including heterogeneities within and across subregions/animals toward enhancing our understanding of the ionic basis of physiology and plasticity in DG granule cells. Such systematic characterizations would provide insights about differential expression of different ion channels in different subregions and their roles under physiological and pathological conditions. The absence of such systematic characterization of ion channel expression profiles could result in deleterious generalizations that assume different subregions of the DG in different species and different strains to be endowed with similar sets of ion channels and similar signature electrophysiological properties. Finally, given the expression of adult neurogenesis in the dentate gyrus (Aimone et al., 2014; Altman & Das, 1965; Deng et al., 2010; Eriksson et al., 1998; Kropff et al., 2015), such systematic studies should also explore ion‐channel gradients and degeneracy in adult‐born granule cells of different ages along these anatomical axes in different species of different strains.

Future computational models of DG neuronal function should employ morphologically realistic models (Beining et al., 2017), with reconstructions obtained from specific subregions and sectors of the dentate gyrus. These models should incorporate all ion channels characterized from specific neuronal subtypes from the individual subregions and sectors, mentioned above. These structural and biophysical details should be employed to construct different populations of models for the distinct DG subregions and sectors. These population of neuronal models could then be employed to understand the specific impact of individual ion channels on the different measurements, and the specific mapping that are prevalent in each of these different subregions. The construction of such models could involve independent multi‐parametric multi‐objective stochastic search (MPMOSS) algorithms (Basak & Narayanan, 2018; Foster et al., 1993; Jain & Narayanan, 2020; Marder & Taylor, 2011; Mishra & Narayanan, 2019; Mittal & Narayanan, 2018; Rathour & Narayanan, 2012a, 2014; Seenivasan & Narayanan, 2020; Taylor et al., 2009) for each of the different subregions and sectors. Such analyses could involve the virtual knockout model strategy where all measurements are repeated across the population of models with each of the several ion channels individually eliminated (Anirudhan & Narayanan, 2015; Basak & Narayanan, 2018, 2020; Jain & Narayanan, 2020; Mishra & Narayanan, 2021; Mukunda & Narayanan, 2017; Rathour & Narayanan, 2014; Sinha & Narayanan, 2015). These approaches could be repeated for adult‐born neurons of different age groups across different subregions, thus extending the analyses to structural heterogeneities as well. Such exhaustive computational analyses would allow to quantitatively explore gradients in physiological and biophysical properties across these different axes and across different ages, also furthering our understanding of ion‐channel degeneracy and of the role of individual ion channels in regulating different measurements in DG neurons across different axes and ages.

### 4.5 **Limitations: Non‐specificities of pharmacological agents**.

A well‐established limitation of pharmacological agents pertains to their non‐specific actions on components other than the intended target. The established non‐specificities of the pharmacological agents employed in this study include those with reference to riluzole on other components (Bryson et al., 1996; Dimitriadi et al., 2013; Doble, 1996; Duprat et al., 2000; Frizzo et al., 2004; Fumagalli et al., 2008), including the ability of riluzole to activate two‐pore domain potassium channels, targeting specific subunits (Duprat et al., 2000) that are known to express and alter excitability of DG granule cells (Reyes et al., 2000; Yarishkin et al., 2014). However, such activation would have resulted in a consistent reduction in input resistance. In our study, we do not observe significant changes in input resistance at RMP (Figure 9b) or at multiple voltages (Figure 9c) with acute treatment with riluzole. In addition, we also do not observe significant changes in maximal impedance amplitude, another measurement that is sensitive to changes in leak channels (Narayanan & Johnston, 2008; Rathour & Narayanan, 2012a; Zemankovics et al., 2010), across different voltages (Figure 9e,f). Although these measurements do not completely rule out the possibility that two‐pore domain potassium channels were activated by riluzole treatment, they provide lines of evidence that riluzole treatment did not result in significant changes to measurements that are sensitive to such activation.

There are known nonspecificities of ZD7288 (Chen, 2004; Chevaleyre & Castillo, 2002; Sanchez‐Alonso et al., 2008) and Ba^2+^ (Zhou et al., 2012). However, in our study, we have employed a constellation of physiological measurements (in conjunction with the pharmacological agents), spanning specific ranges of voltages. As treatment with these agents provide signature electrophysiological changes that are consistent with the blockade of the ion channel under consideration, we believe that our experiments provide clear lines of evidence for the roles of each of the different ion channels in DG granule cells. Specifically, we observe that treatment with ZD7288 or BaCl_2_ results in an enhancement of subthreshold excitability, with the quantum of enhancement shown to be higher in hyperpolarized voltages (Figure 2c for ZD7288, Figure 4c for BaCl_2_). These observations are consistent with both HCN and K_ir_ channels being hyperpolarization‐activated ion channels. In addition, BaCl_2_ and ZD7288 have been shown to be independently blocking K_ir_ and HCN channels, respectively, across different cell types (Bal & Oertel, 2000; Baruscotti et al., 2010; Borin et al., 2014; Datunashvili et al., 2018; Day et al., 2005; Dickson et al., 2000; Hogg et al., 2001; Lee & Ishida, 2007; Li et al., 2017; Ma et al., 2003). In this study, we provide experimental evidence on the independent impacts of ZD7288 and BaCl_2_ in enhancing DG granule cell excitability (Figures 5 and 6). Thus, the constellation of physiological measurements, especially measured at different voltages, and the pharmacological delineation provide several lines of evidence that the action of ZD7288 and BaCl_2_ are on HCN and K_ir_ channels, respectively, on DG granule cells. In addition, there are corroborative lines of evidence from other studies pointing to the expression of each of these channels in DG granule cells (Artinian et al., 2011; Beining et al., 2017; Bender et al., 2003; Crill, 1996; Ellerkmann et al., 2003; Epsztein et al., 2010; Kress et al., 2010; Krueppel et al., 2011; Stegen et al., 2009, 2012; Surges et al., 2012; Young et al., 2009).

In conclusion, our results provide experimental evidence for a heterogeneous many‐to‐many mapping between ion channels and single‐neuron intrinsic properties, thereby electrophysiologically testing the postulate on the expression of ion‐channel degeneracy in DG granule cells. Our results emphasize the many‐to‐many mapping between structural components and functional outcomes and the consequent degeneracy in emergence of function to be central to biological function. These observations underscore the need to account for degeneracy in functional outcomes, and the consequent heterogeneities in the structural components that yielded these similar functions in physiological and pathological studies of the nervous system.

## CONFLICT OF INTERESTS

The authors declare no competing interests.

## AUTHOR CONTRIBUTIONS

Conceptualization, P.M. and R.N.; Methodology, P.M. and R.N.; Investigation, P.M.; Writing – Original Draft, P.M. and R.N.; Writing – Review & Editing, P.M. and R.N.; Funding Acquisition, P.M. and R.N.; Resources, R.N.; Supervision, R.N.

## Data Availability

The published article includes all data generated or analyzed during this study. Any additional data or code requirements will be fulfilled by the corresponding author upon reasonable request.
